# A comparative analysis of gene and protein expression in chronic and acute models of photoreceptor degeneration in adult zebrafish

**DOI:** 10.3389/fcell.2023.1233269

**Published:** 2023-09-07

**Authors:** Ashley C. Kramer, Justin Carthage, Yasmeen Berry, Katherine Gurdziel, Tiffany A. Cook, Ryan Thummel

**Affiliations:** ^1^ Department of Ophthalmology, Visual and Anatomical Sciences, Wayne State University School of Medicine, Detroit, MI, United States; ^2^ Genomic Sciences Core, Wayne State University, Detroit, MI, United States; ^3^ Center for Molecular Medicine and Genetics, Wayne State University School of Medicine, Detroit, MI, United States

**Keywords:** photoreceptor degeneration, chronic light, microglia, Müller glia, stem cell, rod precursor, zebrafish, 3′mRNA-seq

## Abstract

**Background:** Adult zebrafish are capable of photoreceptor (PR) regeneration following acute phototoxic lesion (AL). We developed a chronic low light (CLL) exposure model that more accurately reflects chronic PR degeneration observed in many human retinal diseases.

**Methods:** Here, we characterize the morphological and transcriptomic changes associated with acute and chronic models of PR degeneration at 8 time-points over a 28-day window using immunohistochemistry and 3′mRNA-seq.

**Results:** We first observed a differential sensitivity of rod and cone PRs to CLL. Next, we found no evidence for Müller glia (MG) gliosis or regenerative cell-cycle re-entry in the CLL model, which is in contrast to the robust gliosis and proliferative response from resident MG in the AL model. Differential responses of microglia between the models was also observed. Transcriptomic comparisons between the models revealed gene-specific networks of PR regeneration and degeneration, including genes that are activated under conditions of chronic PR stress. Finally, we showed that CLL is at least partially reversible, allowing for rod and cone outer segment outgrowth and replacement of rod cell nuclei via an apparent upregulation of the existing rod neurogenesis mechanism.

**Discussion:** Collectively, these data provide a direct comparison of the morphological and transcriptomic PR degeneration and regeneration models in zebrafish.

## 1 Introduction

Neurodegenerative diseases of the retina affect approximately 12 million people every year in the United States, and is consistently increasing (Flaxman et al., 2017; CDC, 2022). These include rare, early-onset congenital diseases of photoreceptor (PR) degeneration (e.g., retinitis pigmentosa, Stargardt disease, congenital night blindness) (Allikmets et al., 1997; Hartong et al., 2006; Berger et al., 2010; Regus-Leidig et al., 2014; Verbakel et al., 2018; García Bohórquez et al., 2021) and more prevalent, late-onset diseases (e.g., age-related macular degeneration, glaucoma, diabetic retinopathy) (Almasieh et al., 2012; Kelbsch et al., 2016; Mitchell et al., 2018; Ali et al., 2020; Antonetti et al., 2021; Saadane et al., 2021). These diseases exhibit dysfunction and progressive death of PRs and other retinal cell types, eventually leading to partial or complete vision loss. Mammals largely lack the capacity to regenerate lost neurons, limiting therapeutic options for these patients. However, teleost fish, some amphibians, and embryonic chicks retain the capacity for retinal regeneration, making them valuable models to develop and inspire new therapeutic approaches (Lamba et al., 2008; Jorstad et al., 2017; Todd et al., 2022).

Adult zebrafish have been highly utilized as a model to better understand the cellular and molecular events during retinal regeneration. A variety of damage paradigms have been characterized in zebrafish, including acute phototoxic lesion, various neurotoxins, laser, and physical damage (Vihtelic and Hyde, 2000; Wu et al., 2001; Vihtelic et al., 2006; Thomas et al., 2012; Hamon et al., 2016; Zhang et al., 2016). These paradigms highlight the resilience and versatility of the zebrafish retina to respond to different damage events and regenerate the neuronal cell types that are lost. The last two decades of characterizing these damage paradigms have revealed multiple key cellular events and genetic pathways that are essential to retinal regeneration in zebrafish. For example, multiple groups revealed that the stem cell responsible for the regenerative capacity of the zebrafish retina is the Müller glia cell (MG) (Fausett and Goldman, 2006; Bernardos et al., 2007; Fimbel et al., 2007; Thummel et al., 2008b). In the absence of injury, MG function in various glial support roles, including structural support, paracrine signaling, neurotransmitter recycling, and water homeostasis (Vecino et al., 2016; Bejarano-Escobar et al., 2017; Reichenbach and Bringmann, 2020). However, upon acute and significant damage to the retinal neurons, zebrafish MG upregulate neuroprotective programs of gliosis and undergo an asymmetric cell division, generating one daughter cell that retains its innate MG function, and one that will become a multipotent MG-derived progenitor cell (MGPC). MGPCs then expand through symmetric divisions, forming clusters of progenitor cells that go on to replace lost retinal cell types (Kassen et al., 2007; Thummel et al., 2008a; Nagashima et al., 2013; Lenkowski and Raymond, 2014). In addition, several groups have revealed key molecular pathways required for retinal regeneration, including Ascl1a, Notch, FGF, Stat3, and Pax6, to name a few (Thummel et al., 2008a; Ramachandran et al., 2011; Nelson et al., 2012; Taylor et al., 2015; Wan and Goldman, 2017). Importantly, groups working in mouse models of retinal degeneration have started to translate some of these molecular findings for targeted therapeutic interventions in the mammalian model (Jorstad et al., 2017; Todd et al., 2022).

A common model to study MG-mediated retinal regeneration in adult zebrafish involves a short pulse of high intensity light (∼100,000 lux), followed by 3–4 days of bright light exposure (∼10,000 lux). This elicits a massive loss of rod and cone PRs, followed by full retinal recovery 28 days after initial light exposure (Vihtelic and Hyde, 2000; Nelson et al., 2013; Kramer et al., 2021). Remarkably, the cellular and genetic events that occur during regeneration are tightly regulated in defined windows of time. For example, initial MG gliosis occurs at 18–24 h after light onset (hpl), as observed by the upregulation of GFAP in the outer nuclear layer (Thomas et al., 2016; Ranski et al., 2018; Kramer et al., 2021). PCNA localization demonstrates that MG re-enter the cell cycle at 36 hpl, and that large MGPC clusters form and migrate to the outer nuclear layer (ONL) from 72 to 96 hpl. This is also the window for the peak expression of 4c4+ microglia in the outer retina, which clear the debris of dead PR outer segments. Finally, markers for rod and cone PRs reveal that new photoreceptor differentiation begins from 5 to 10 days after light onset (dpl) (Thummel et al., 2008a; Kramer et al., 2021). Over the next few weeks, MGPC proliferation ends, PR outer segments continue to expand, microglia are resolved to baseline activity, and MG return to normal homeostatic function. Recently, we published an in-depth transcriptomic analysis of this acute light (AL) damage model that corresponds to each of these key morphological events in retinal regeneration. This included transcriptomic analysis of 8 defined time-points during the regeneration process, from undamaged control retinas to 28 days post-light onset (Kramer et al., 2021). This work complements a recent multi-species comparative analysis dataset using scRNA-seq from retinas harvested between 0–72 hpl (Hoang et al., 2020), and reveals new potential genetic targets for retinal regeneration following acute damage.

Despite the success of acute damage models in revealing molecular pathways that promote and regulate retinal regeneration, they do not accurately reflect the slow PR degeneration seen in most human retinal disease processes. As such, we and others have developed chronic models of retinal damage (Iribarne et al., 2019; Santhanam et al., 2020; Turkalj et al., 2021). A preliminary analysis of our chronic low light (CLL) model indicated that cones were more resistant to CLL than rods and that a longer, less intense light model failed to trigger the classic MG-mediated gliosis and regeneration mechanism observed in acute damage models (Turkalj et al., 2021). In this follow-up work, we performed an in-depth morphological and transcriptomic analysis of the CLL model, which allowed us to directly compare it to the AL model at the exact same 8 time-points that were previously defined. Here, we report new insights into the differential resilience of cone vs. rod PRs to damage, and identify novel dynamics in putative PR protection pathways. In addition, we show that CLL does not elicit MG gliosis or robust cell cycle re-entry. Next, comparative transcriptomic analysis of PR degeneration and regeneration in the chronic and acute models, respectively, revealed inverse patterns of gene expression among conserved transcriptional pathways in PR function and development. It also revealed genes that could play a role in PR survival under chronic stress, including dynamic changes in cone opsin expression and apparent microglia activation and resolution. Lastly, we show that PR outer segment damage in the CLL model is at least partially reversible, opening a new opportunity for investigation into PR outer segment recovery. Collectively, these data provide the field with new potential directions for studies aimed at understanding the distinctions between regenerative and non-regenerative retinal model systems.

## 2 Materials and methods

### 2.1 Husbandry

All experiments utilized adult *albino* (*alb*) zebrafish (6–9 months of age). Zebrafish were fed three times daily a combination of brine shrimp and flake food. The fish were maintained under a closed water circulation system with a standard circadian light cycle which consists of 14 h of light (∼250 lux) and 10 h of dark at 28.5°C (Westerfield, 2000). The Institutional Animal Care and Use Committee at Wayne State University approved all procedures used in this study (Protocol # 22-02-4412).

### 2.2 Light treatment protocol

**FIGURE 1:**
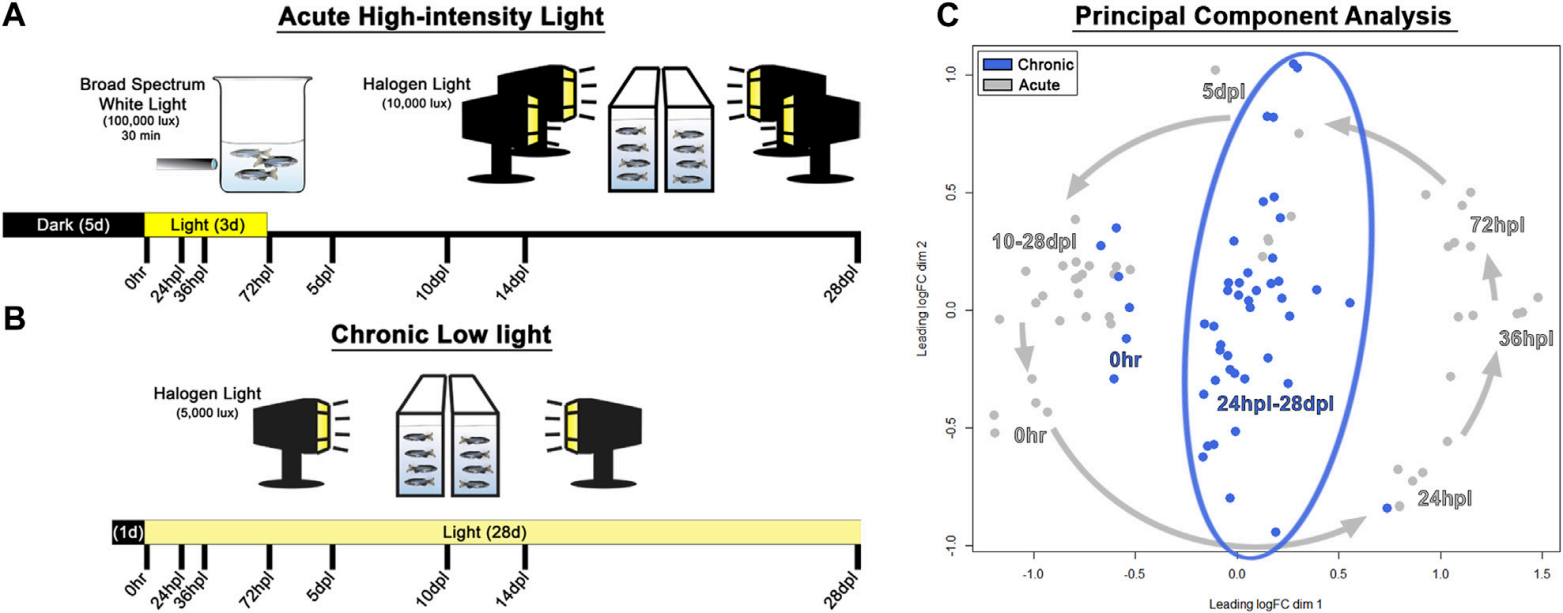
Methods and principal component analysis. **(A)** Experimental design used for acute phototoxic lesion, with tissue collection time-points along the bottom designated in hours/days post-light onset (hpl/dpl). **(B)** Chronic low light lesion model set-up demonstrating reduced intensity of the light lesion protocol with the same tissue collection time-points. The initial 100,000 lux step was removed, and the secondary stage was reduced in lux by 50% (compared with the AL model) and prolonged for 28 days. **(C)** Principal component analysis of the top 200 genes which maximized separation of the dark-adapted, 0 h baseline controls for both chronic low and acute light datasets. Acute light damage samples are displayed in grey, and chronic low light datasets are blue. The grey arrows represent the temporal progression trajectory of the tissue collection time-points. For the chronic low light samples, there was no clear temporal progression of the transcriptional landscape as there was in the acute light regeneration dataset, rather, tissue collection time-points post 0 h baseline were scattered in the middle of the plot.

The acute light treatment model used for these experiments was exactly as described previously (Kramer et al., 2021). For the chronic low light treatment protocol, fish were only dark adapted for 24-h prior to initiation of the light exposure (vs. 5 days for the acute light treatment), so as to only partially sensitize the animals to the subsequent light. The “0 h” sample set was harvested immediately after dark adaptation. The remaining fish were subsequently exposed to 28 days of continuous exposure to two 250 W halogen bulbs (Woods Home Products, Carrollton, Georgia; Figure 1B). Two rounds of light treatments were conducted with a total of *n* = 3 animals per tissue collection time-point, resulting in an overall *n* = 6. The tissue collection time-points were as follows: a non-light treated control group (0 h), 24 h post light onset (hpl), 36 hpl, 72 hpl, 5 days post light onset (dpl), 10 dpl, 14 dpl, and 28 dpl.

### 2.3 Immunohistochemistry

Right eyes were removed from euthanized animals then transferred into a 9:1 ethanolic formaldehyde solution overnight at 4°C. Next, eyes were washed in 5% sucrose/1X Phosphate Buffered Saline (PBS) at room temperature (RT) for 1–2 h, and then transferred to 30% sucrose/1XPBS overnight at 4°C. The following day, eyes were transferred to a 1:1 solution of 30% sucrose/1XPBS and Tissue Freezing Medium (General Data, Cincinnati, OH) overnight at 4°C. Next, eyes were embedded in 100% TFM and stored at −80°C, prior to cryosectioning at a thickness of 16 μm. Sections were transferred to Superfrost Plus glass microscope slides (Fisher Scientific, Waltham, MA) and dried on a slider warmer at 55°C for 1–2 h before being stored at −80°C.

Stored slides were re-warmed for 20 min on a 55°C slide warmer. Tissue was rehydrated with 1xPBS for 20 min, and then incubated in blocking solution (2% normal goat serum, 0.2% Triton X-100, and 1% dimethyl sulfoxide (DMSO) in 1XPBS) at RT for 1 h. Slides were incubated overnight at RT with primary antibodies diluted in blocking solution. The next day, sections were washed 3% × 0.05% Tween-20 in 1XPBS (1XPBT). Secondary antibodies along with the nuclear stain TO-PRO-3 (Life Technologies, Grand Island, NY) were diluted 1:500 in 1XPBT. The tissue was washed with 1XPBT after incubation and mounted using Prolong Gold Antifade Reagent (Molecular Probes, Eugene, OR).

Primary antibodies used included: rabbit anti-Rhodopsin antisera (gift from David Hyde; 1:5,000), mouse anti-PCNA (Sigma; 1:1,000), rabbit anti-Blue Opsin (gift from David Hyde; 1:500), rabbit anti-UV Opsin (gift from David Hyde; 1:1,000), rabbit anti-Green Opsin (gift from David Hyde; 1:500), mouse anti-zpr-1 (Zebrafish International Resource Center; 1:200) rabbit anti-GFAP (DakoCytomation; 1:500), mouse anti-4C4 (gift from Peter Hitchcock; 1:250), mouse anti-HuC/D (Life Technologies, Grand Island, NY; 1:50). Secondary antibodies used were all AlexaFluor-conjugated 488 and 594 anti-primary (1:500, Life Technologies, Grand Island, NY).

### 2.4 TUNEL analysis

A terminal deoxynucleotidyl transferase dUTP nick end labelling (TUNEL) assay was performed to detect DNA damage. Slides containing sectioned tissue were removed from −80°C and warmed for 20 min on a 55°C slide warmer. Sections were rehydrated for 20 min in 1XPBS. Slides were then incubated with ApoTag^®^ Equillibration Buffer (EMD Millipore, Darmstadt, Germany) for 10 min at 37°C. The TUNEL reaction mix was prepared using the TdT dNTP mix (R&D Systems, Minneapolis, MN) and TdT enzyme (Takara Bio, Japan, Korea) according to manufacturer protocol, and slides were incubated for 2 h at 37°C. Slides were then washed in 2X SSC buffer for 15 min at RT, followed by 2 washes with 1XPBS. Slides were then labeled with an AlexaFlour 488 Streptavidin secondary antibody (1:200; Life Technologies, Grand Island, NY) for detection of incorporated dNTPs and TO-PRO-3 (1:500) in 0.1% Tween-20 in 1XPBS for 1 h at RT. Slides were then washed 3 x in 0.1% Tween-20 and a final wash in 1XPBS before being mounted and cover-slipped with Prolong Gold prior to imaging.

### 2.5 Confocal microscopy and image quantification

A Lecia TCS SP8 confocal microscope was used to acquire all images. Images were taken of the central dorsal retina on a singular plane using the same exposure and gain. Next, images were processed using ImageJ to isolate the fluorescence channel of interest, and the mean fluorescence intensity within a given ROI was quantified (*n* = 5-6 images quantified per marker). Quantified fluorescent intensities were normalized to the 0 h baseline intensity. PR outer segment lengths were also quantified using ImageJ (Schneider et al., 2012). Three measurements were taken at different areas and averaged for each image quantified. The length measurements were taken in arbitrary units and normalized to the 0 h baseline. For the TUNEL assay, 4c4 staining of microglia, PCNA staining, and cone cell quantification, cells were counted by hand over a 300 µm linear distance in the central dorsal retina. For ONL nuclear counts, TO-PRO-3 positive cells in the ONL proper were quantified (i.e., within the outer limiting membrane). Given that the vast majority of the ONL nuclei are rod PR nuclei, this is commonly used as a proxy for rod PR numbers; however, it should be noted that the ONL also contains UV cone nuclei (Raymond and Barthel, 2004; Lagman et al., 2015). Nuclei for the rest of the cone PRs lie outside of the outer limiting membrane (Raymond and Barthel, 2004; Lagman et al., 2015). For all quantification methods, a one-way ANOVA was used to determine statistical significance across the time-course, followed by a *post hoc* Tukey test to determine any statistical differences between any time-points.

### 2.6 Quantification of microglia area

RGB confocal images were split into the respective channel image components using ImageJ 1.53k (Schneider et al., 2012). An automated image processing pipeline was created using CellProfiler 4.2.4 (CellProfiler Image Analysis Software, RRID:SCR_007358) (Carpenter et al., 2006; Jones et al., 2008; Jones et al., 2009; Kamentsky et al., 2011). A region of interest was manually defined in each confocal image based on TO-PRO-3 signal. This region was then used to mask channel images used for subsequent analyses. Green channel images were converted to grayscale, and the 4c4 fluorescence signal was enhanced using the EnhanceorSuppressFeatures module with neurites feature type and line structures enhancement method. Cell bodies were then identified based on these enhanced 4c4 grayscale images as primary objects, using global otsu two-class thresholding strategy. Overlapping objects were distinguished based on intensity, and dividing lines drawn by propagation. The area of objects was measured in pixels using the MeasureObjectSizeShape module, and reported on an exported spreadsheet. Subsequent statistical analyses were performed using GraphPad Prism 9.

### 2.7 cDNA library preparation and 3′RNA-Seq analysis from retinal RNA

Retinas from left eyes of animals were dissected and processed for RNA isolation as individual biological replicates for each of the eight tissue collection time-points as previously described (Kramer et al., 2021). Notably, with the retinal harvest technique, there is a possibility of some RPE and vitreous inclusion in the sample, however the optic nerve is excised. A set of non-dark adapted 0 h control age-matched adult *albino* control retinas were also harvested to use as a naïve control group to compare dark adaptations in the AL and CLL models. This purified RNA was submitted to the Genome Sciences Core at Wayne State University for RNA QC, cDNA library preparation, and sequencing. Sequencing libraries were generated from 250 ng of total RNA using Lexogen’s Quantseq 3′mRNA-Seq Library Prep Kit FWD. Sequencing was performed on a NovaSeq (minimum of 5Mreads per sample). Reads were aligned to the zebrafish genome (Build dR10) (Dobin et al., 2013) and tabulated for each gene region (Anders et al., 2015).

Differential gene expression analysis was used to compare transcriptome changes between conditions (6 replicates per condition across two separate experiments) (Robinson et al., 2010). In addition, separate pairwise comparisons were also performed for the 0–5 dpl samples in the CLL dataset and the 5–10 dpl samples in the AL data set for subsequent gene ontology analysis and comparison was performed with the open-source GOrilla platform (Eden et al., 2007; Eden et al., 2009) using a fold change cutoff of greater than 2-fold increase or decrease in gene expression with an FDR of <0.05. Redundant terms were consolidated and visualized in the “Reduce and Visualize Gene Ontology” (REVIGO) platform as an output (Supek et al., 2011). From the REVIGO output, the. tsv file was recovered from the “tree map view,” and formatted for input into the 2D pie chart gene ontology visualization software CirGO to visually represent the GO categories (Kuznetsova et al., 2019). The 13 overlapping genes identified in Figure 6 were input into the STRING platform to asses literature-based functional protein association network relationships (Szklarczyk et al., 2015). Mapped reads for the entire dataset can be found in Supplementary Table S1. The datasets presented in this study can be found in the NCBI Gene Expression Omnibus, accession no: GSE233896, and the associated metadata for this submission can be found in Supplementary Table S2.

## 3 Results

### 3.1 Principal component analysis revealed absence of a clear transcriptional trajectory in the chronic vs. acute light lesion models

To characterize and compare the response of the zebrafish retina under chronic low light (CLL) exposure to the classic PR ablative acute light (AL) damage paradigm (Vihtelic and Hyde, 2000; Thummel et al., 2008a; Kramer et al., 2021), we subjected adult *albino* zebrafish to either 3 days of intense AL followed by 25 days of recovery, or 28-day of CLL (Figures 1A, B). Eye tissue was harvested at the same 8 time-points throughout both light treatment time-courses, with whole right eyes from each animal processed for immunohistochemistry, and dissected retinas from left eyes harvested for single biological replicate transcriptomic studies. The 8 time-points chosen for investigation were largely governed by our previous detailed characterization of the AL damage model (Kramer et al., 2021), which allowed us to directly compare the CLL and AL models in this follow-up work.

Principal component analysis (PCA) was performed on the top 200 genes which maximized separation of the dark-adapted 0 h baseline controls for both the chronic and acute light datasets, such that the distance between each pair of samples represented the root-mean-square deviation for these top genes. This analysis revealed that AL treatment resulted in a counterclockwise trajectory that followed a chronological progression, with early damage time-points (24–72 hpl) diverging most significantly from 0 h baseline controls, followed by a gradual return to near the 0 h baseline at later time-points (10–28 dpl; Figure 1C, grey arrows), which is consistent with the full recovery observed in this model. In the CLL model, 1-day dark-adapted transcriptomes of the samples clustered more closely with the grouping of 10–28 dpl samples in the AL treatment, rather than the 5-day dark-adapted samples in that dataset (Figure 1C), suggesting that the transcriptomes of the CLL-dark adapted retinas align more closely to that of newly regenerated retinas than to 5-day dark-adapted retinas. In addition, after the initiation of light, we observed a considerably higher variability in the time-point clustering in the CLL than in the AL. Interestingly, the CLL time-points were arranged in a linear fashion within the surrounding AL time-points, with the majority of early time-points in the CLL clustered toward the bottom and the majority of the later time-points near the top (Figure 1C).

### 3.2 CLL exposure leads to rapid but stable changes in cone photoreceptor morphology, in contrast to complete cone cell ablation in the AL exposure model

**FIGURE 2:**
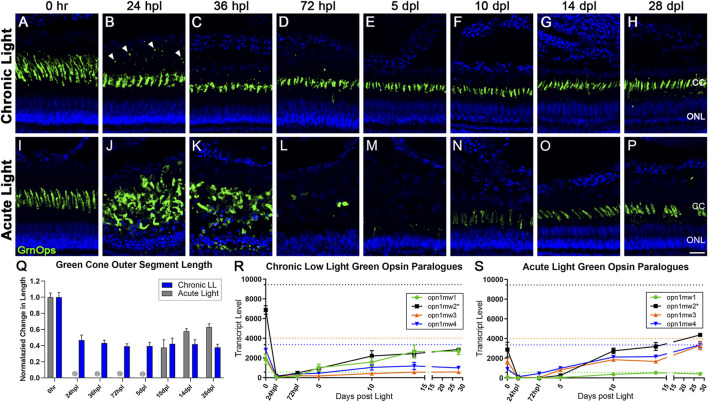
Differential morphology and *opsin* expression from cone photoreceptors in CLL and AL models. Green cone PRs were immunolabeled with anti-green opsin antisera (green) and nuclei were stained with TO-PRO-3 (blue). **(A–H)** Green opsin-positive cone PRs remained intact in response to the chronic light damage, but exhibited outer segment truncation over a 28-day CLL exposure. **(B)** At 24 hpl we observed a significant truncation of green cone PR outer segments (OS) as well as small puncta of immuno-positive debris in the outer retina. **(C,D)** At 36 and 72 hpl we observed a gradual reduction of the debris in the outer retina, but a continued truncation of green cone PR outer segments. **(E–H)** From 5–28 dpl green cone PRs remained intact, as evidenced by their cone cell nuclei and continued green opsin expression, but their outer segments remained truncated throughout the time-course. **(I–P)** Green cone PRs exhibited a classic degeneration and regeneration response to acute light damage. **(J)** At 24 hpl, cone OS were hypertrophied and ONL nuclei were pyknotic. **(L)** At 72 hpl, cone OS and their nuclei were completely absent. **(N)** Evidence of newly formed green cones was present by 10dpl. **(Q)** Green cone outer segment length quantified with ImageJ normalized to 0 h baseline in chronic vs. acute damage models (*n* = 5–7). For AL images, length was not calculated for 24 hpl-5 dpl as the cones were dying or dead. The OS length loss from the 0 h baseline was significant (*p* < 0.0001) starting at 24 hpl, and continuing for every consecutive time interval in the chronic low light samples. **(R,S)** Gene expression changes in all four paralogues of the green opsin gene (*opn1mw1-4*) displayed as transcript pseudocounts from 3′mRNA-seq of individual retinas for **(R)** CLL and **(S)** AL. Dotted lines represent baseline non-dark-adapted naïve control transcript levels. The asterisk in the key for *opn1mw2* denotes the dominantly expressed paralogue at both the naïve and dark-adapted baselines in each model. **(R)** In CLL, the non-dominantly expressed green opsin paralogues *opn1mw3/4* throughout the time-course*,* while the lowest expressed paralogue (green) recovers to the same exact level as the dominant opsin (black). **(S)** As time progresses during regeneration in the AL model, however, the dominantly expressed green opsin (black) exhibits a trend of recovery, along with two additional paralogues *mw3/4* (orange and blue), while the lowest expressed opsin, *mw1* remains low (green). (CC = green cone cell outer segment, ONL = outer nuclear layer; scale bar in panel P = 5 µm).

We next performed a morphological analysis of cone PRs in response to CLL (Figures 2A–H) and AL (Figures 2I–P). In the CLL model, green (medium wavelength, mw) cone outer segments (mwCOS) exhibited a ∼50% truncation in length within the first 24 h of light exposure, which was maintained through the remainder of the time-course (*p* < 0.0001, Figures 2B–H, Q). Sparse opsin-positive debris appeared posterior to the cone OS boundary at 24 hpl, but subsided by 72 hpl (Figures 2B–H, Q). UV and blue cones exhibited a similar pattern of OS truncation (Supplementary Figure S1), although short-wavelength (blue) cones appeared to be the least impacted by the CLL damage paradigm, as evidenced by a minimal debris field at 24 hpl, and less severe OS truncation (60%–80% normal lengths; Supplementary Figures S1M, T). In contrast, consistent with our previous findings (Kramer et al., 2021), in the AL model, green cones were rapidly ablated by 36 hpl (hours post light onset; Figure 2K). The resultant debris field was largely cleared by 72 hpl (Figure 2L), and new cones emerged by 10 dpl (days post light onset; Figure 2N).

Analysis for red/green double cone somas in the CLL model using the anti-Arrestin3 (Arr3) antibody, zpr-1, confirmed that double cones remained intact throughout the CLL time-course (Supplementary Figure S2). In addition, quantification of UV, blue, and green opsin outer segments confirmed that these cones also remained intact during the CLL time-course (Supplementary Figure S3). Nevertheless, we observed a translocation of Arr3 from the cone soma to both the cone OS and pedicles between 24–36 hpl (Supplementary Figure S2A–C), and the pedicle expression was sustained throughout the CLL time-course (Supplementary Figure S2H). Finally, the inner retina appeared unaffected in the CLL model based on morphology and HuC/D staining of amacrine and ganglion cells (Supplementary Figure S4).

Next, we compared the transcripts of the cone PR opsins in the CLL and AL models. We previously demonstrated that the 5-day dark adaptation utilized in the AL model, which sensitizes PRs to light, decreased all PR opsin transcript levels (Kramer et al., 2021). Here we show that the 1-day dark adaptation used in the CLL model also decreased most PR opsin levels, but not to the degree observed in the AL model (Figures 2R, S). In the AL model, transcripts of the dominant Green Opsin paralog *opn1mw2* were reduced to zero by 24 hpl, due to the ablation of these PRs (Figure 2S). Transcript levels increased from 5 to 10 dpl as progenitors differentiated into new double cones, and increased even further by 28 dpl, exceeding the expression level of the 0 h control retinas (Figure 2S). Surprisingly, in the CLL model, *opn1mw2* transcripts were also reduced to near zero by 24 hpl, even though these PRs remained intact (Figures 2B, S. From this point, *opn1mw2* transcripts slowly rose throughout the time-course, but never reached even 50% of the baseline level of the 0 h control retinas (Figure 2S).

### 3.3 Differential medium wavelength opsin gene regulation in CLL vs. AL models

It should also be noted that Green Opsin has four paralogues in zebrafish (*opn1mw1, opn1mw2, opn1mw3, opn1mw4*). In both damage models, all four paralogues were highly expressed in naïve retinas, with *opn1mw2* exhibiting the highest expression levels at the dark adapted 0 h baseline (Figures 2R, S; black). However, the different dark adaptation durations of 1 day in the CLL model, and 5 days in the AL model, revealed surprising transcriptional consequences. In the CLL model, the least expressed opsin at naïve control baseline levels, *opn1mw1,* exhibited an increase in transcript levels of nearly 10-fold during the 1-day dark adaptation (Figure 2R, green), the only opsin across both models to increase with dark adaptation. Next, following an initial decrease in expression at 24 hpl, we observed that *opn1mw1* transcripts increased significantly, surpassing the naïve control baseline levels to match the levels of the dominantly expressed paralogue *opn1mw2* (Figure 2R, green), which remained well below its baseline level, along with the other two paralogues, *opn1mw3* and *opn1mw4.* The transcriptional kinetics of these paralogues follows a completely distinct trajectory in the AL model as compared to the CLL model. In the AL model, all 4 paralogues showed a significant decrease in transcript levels during the 5-day dark adaptation (Figure 2S). Interestingly, *opn1mw3* and *opn1mw4* returned to near-naïve levels by 28 dpl, whereas the dominant opsin, *opn1mw2*, only returned to dark-adapted control levels (Figure 2S). In the AL model, the *opn1mw1* paralogue, which was the most active from baseline in the CLL model, had a very minor response, just reaching its low naïve control baseline level. These data suggest regulated differential control of opsin expression in the two damage paradigms.

### 3.4 CLL exposure leads to slow rod photoreceptor degeneration, whereas rod photoreceptors are destroyed and replaced in the AL model

**FIGURE 3:**
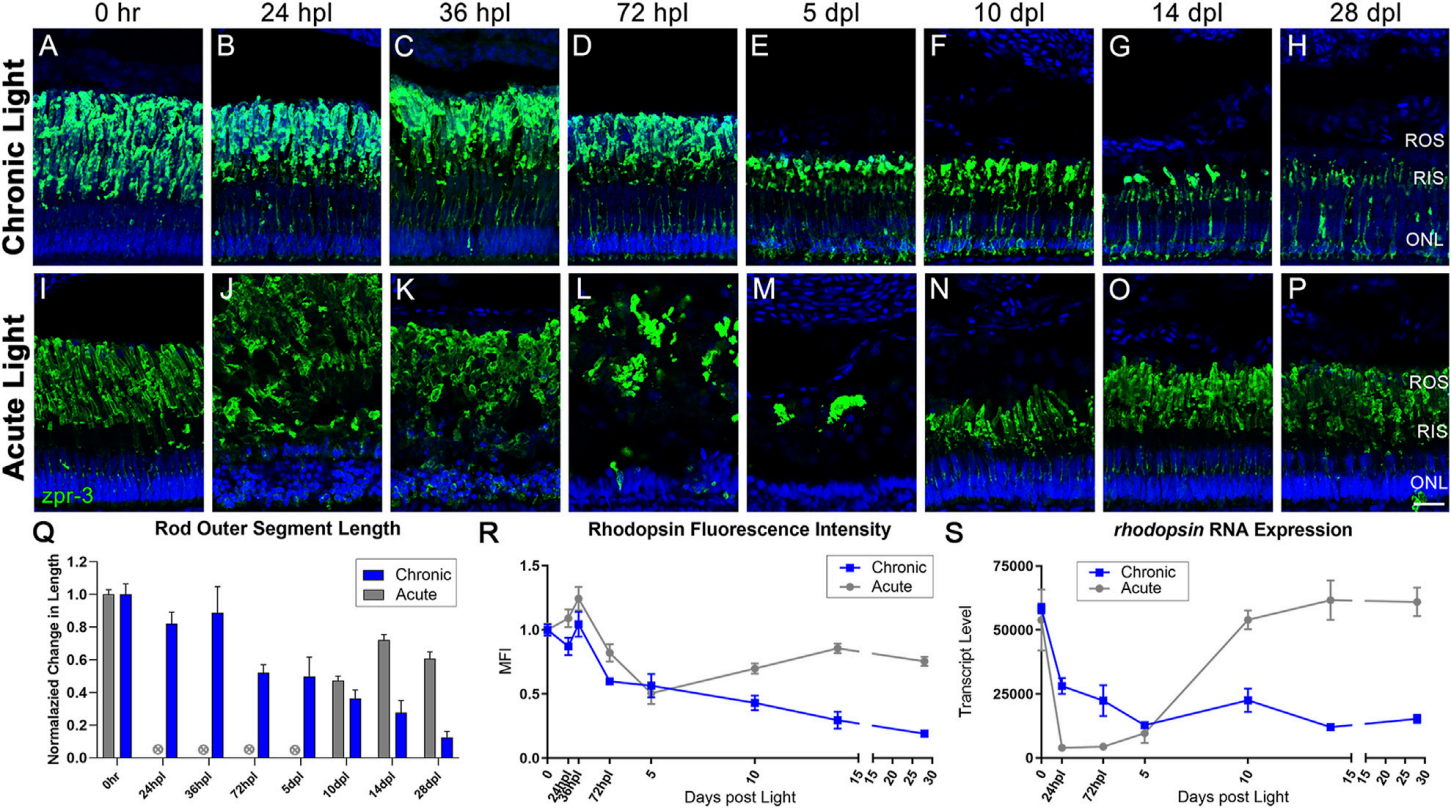
CLL exposure leads to slow rod photoreceptor degeneration, whereas rod photoreceptors are destroyed and replaced in the AL model. Rod PRs were immunolabeled (green) with zpr-3 to mark rod outer segments (ROS) and nuclei were stained blue with TO-PRO-3. **(A–H)** In response to chronic low light exposure, zpr-3 positive ROS displayed a gradual truncation over time until they were almost absent at 28dpl. **(B)** At 24 hpl, zpr-3 expression began to localize in the rod inner segments (RIS). **(C)** At 36 hpl, localization of zpr-3 expression was seen in both the RIS and outer nuclear layer (ONL). **(E)** By 5 dpl and onward, ROS were significantly truncated in length and zpr-3 expression was observed in the perinuclear area, along with a reduction in quantity of ONL nuclei. **(I–P)** Rod PR response to acute light damage. **(J)** At 24 hpl, ROS exhibited significant hypertrophy and ONL nuclei were pyknotic and disorganized. **(M)** At 5 dpl, the ROS debris field was cleared, and evidence of newly formed rods was observed at 10dpl **(N)**. **(Q)** ROS length measured via ImageJ, normalized to 0 h baseline in chronic vs. acute damage models. Each time-point after 72 hpl in the CLL model shows a statistically significant decrease in ROS length as compared to the 0 h baseline (*n* = 5–6; *p* < 0.012–0.001). For AL images, length was not calculated for 24 hpl-5 dpl as the PRs were dying or dead. The final three time-points in the AL model were also statistically significant (*p* < 0.0001). **(R)** Percent change from 0 h in ImageJ-quantified zpr-3 mean fluorescence intensity (MFI) at each time-point. Each time-point 72 hpl and beyond in the CLL model represented a statistically significant MFI as compared to 0hpl in ROS (*p* < 0.003–0.0001). **(S)** Gene expression changes in *rhodopsin* in CLL vs. AL damage models displayed as transcript pseudocounts from 3′mRNA-seq of individual retinas. A steady decrease in *rho* gene expression was observed throughout the time-course in CLL retinas. (ROS = rod outer segments, RIS = rod inner segments, ONL = outer nuclear layer; scale bar in panel P = 5 µm).

Next, we compared the response of rod PRs to CLL and AL exposure by labeling the Rhodopsin protein with the zpr-3 monoclonal antibody, which normally labels rod outer segments (ROS; Figure 3). In contrast to the early, maintained phenotypes for cones in the CLL model, rod PRs showed a gradual damage and loss under CLL conditions. By 72 hpl, we observed the beginning of a progressive truncation of ROS which continued through the end of the time-course, at which point the remaining OS were less than 20% of their normal length (Figures 3D–G, Q; *p* < 0.0001). By 5 dpl we also began to observe a slow loss of nuclei in the ONL over the remainder of the time-course (Figures 3E–H) and reduced *rhodopsin* gene expression (Figure 3S). From 36 hpl forward, 1-4 TUNEL-positive cells were detected within a 300 µm linear distance in the dorsal retina at each time-point, consistent with a slow loss of nuclei on a daily basis (Supplementary Figure S5) (Vihtelic and Hyde, 2000). By 28 days, a ∼55% reduction in ONL nuclei was observed (0 h baseline: 287 nuc ±18.7, 28 dpl: 126.8 nuc ±26.2). We also observed additional morphological consequences of CLL exposure, beginning with faint zpr-3 expression in the rod inner segments (RIS) starting at 24–36 hpl (Figures 3C, D). This RIS misexpression continued to progressively localize to the inner compartments of rod PRs, resulting in robust perinuclear localization of the zpr-3 signal by 10 dpl (Figure 3F). This finding is consistent with aberrancies seen in various mouse models of retinal degeneration and our previous observations (Kong et al., 2006; Li et al., 2020; Turkalj et al., 2021).

All of these observations were in stark contrast to the AL model, where it is well-established that rod PRs die within the first 24–36 hpl (Vihtelic and Hyde, 2000; Nelson et al., 2013; Kramer et al., 2021). This resulted in extreme hypertrophy of the ROS and disorganization of the ONL nuclei at 24–36 hpl (Figures 3J, K) and a dramatic drop in *rhodopsin* transcript levels (Figure 3S). A large opsin-positive debris field was still present at 72 hpl (Figure 3L) but was largely cleared by 5 dpl (Figure 3M). Finally, *rhodopsin* transcript levels increased dramatically from 5 to 10 dpl (Figure 3S), at which point new rod PRs were evident (Figure 3N). Maturation and ROS length increased from 10 to 28 dpl, but never returned to baseline control levels (Figure 3O–Q).

### 3.5 CLL exposure induces a distinctly different microglial response than acute light damage

**FIGURE 4:**
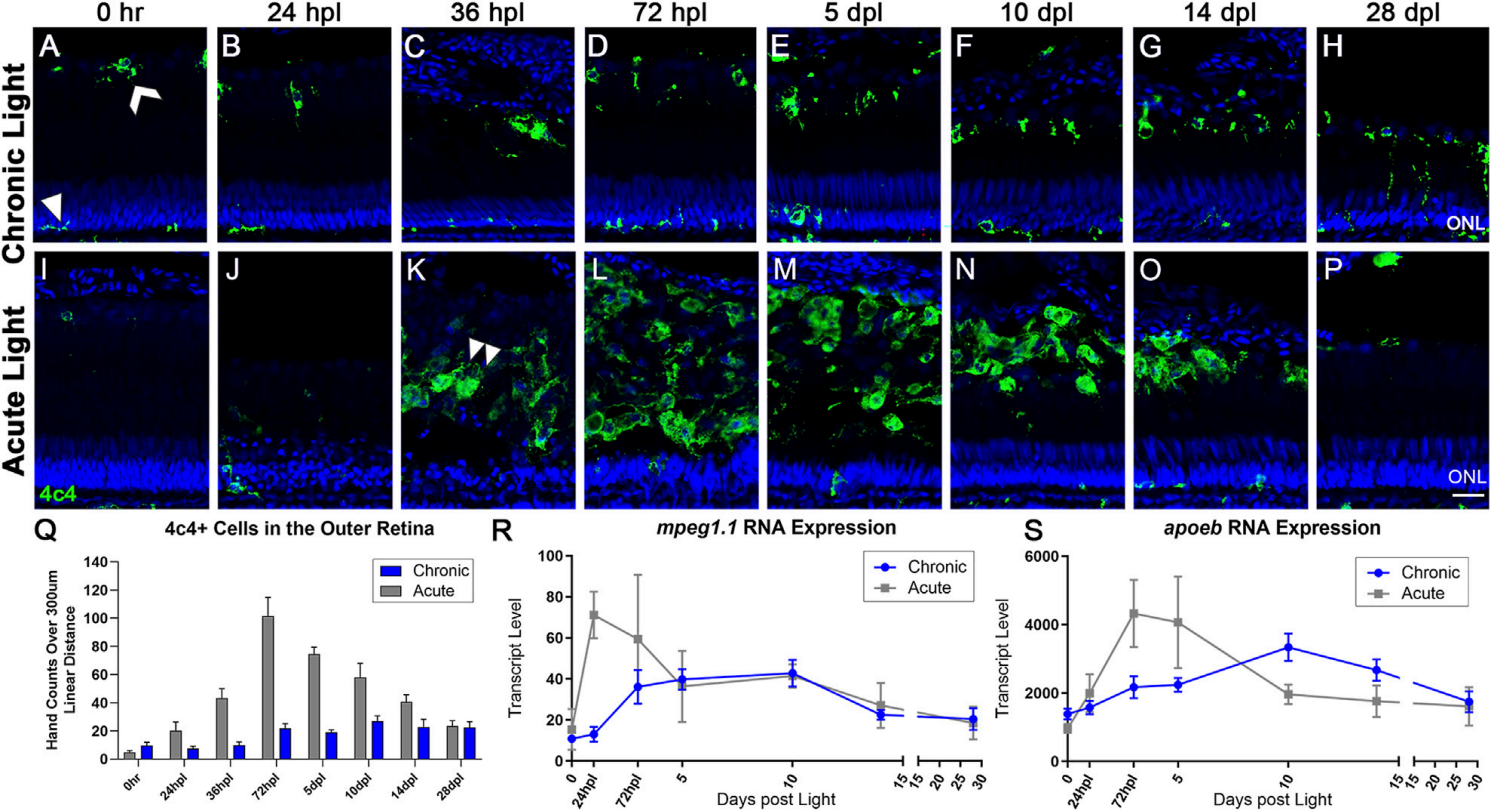
The microglial response to CLL is muted in comparison to AL damage. Anti-4c4 antisera (green) was used to immunolabel microglia and nuclei were stained with TO-PRO-3 (blue). **(A)** At rest, microglia exhibited two distinct morphologies. Within the ONL and plexiform layers, the microglia were ramified with thin projections (white arrowhead). In the outer retina, microglia resided at the outer margin of the ROS/RPE boundary in an activated state, exhibiting a slightly larger morphology with fewer projections (white chevron). **(A–H)** Over 28-day CLL exposure, we observed a slight increase in the microglia inflammatory response in the outer retina. **(D)** At 72 hpl, microglia accumulated at the tips of the truncated ROS (Figure 2). **(H)** At 28 dpl, we also observed some microglia interdigitating with ROS. **(I–P)** Microglia response in the AL damage model. **(K)** Microglial infiltration corresponded directly to peak PR damage in the AL model, with the first evidence of microglia with amoeboid morphology occurring at 36 hpl (double arrowhead). **(M)** The peak of microglial presence in the outer retina occurred at 72 hpl, corresponding to the completion of the debris clearance of cones, and significant consolidation of the ROS debris field. **(Q)** 4c4+ cell hand counts in the outer retina (not including ONL) over a 300 µm linear distance. In the CLL model, the only time-point with significantly higher 4c4+ cells in the outer retina as compared to 0 h is at 10 dpl (*p* < 0.011). In the AL model, there was a significant increase in 4c4+ cells for all time-points from 36 hpl–14 dpl (*p* < 0.041-p<0.001). **(R,S)** Gene expression changes in two microglia-associated genes (*mpeg1.1* and *apoeb*) in CLL vs. AL damage models displayed as transcript pseudocounts from 3′mRNA-seq of individual retinas. In the AL model, we observed an early acute increase in these genes in a peak from 24 hpl to 5 dpl that preceded the observed increase in immunolabeled microglia. In the CLL model, the pattern of gene expression followed a similar pattern, only the peak occurred later, at 10 dpl and the wave was longer in duration. (ONL = outer nuclear layer; scale bar in panel P = 5 µm).

Müller glia/macroglia function together with microglia in a number of developmental, disease, and regeneration contexts (White et al., 2017; Mitchell et al., 2019; Issaka Salia and Mitchell, 2020; Var and Byrd-Jacobs, 2020). In 0 h CLL control retinas, microglia appeared to exhibit two distinct morphologies (Figure 4A; Supplementary Figure S6A). Within the inner and outer plexiform layers, microglia exhibited a ramified morphology with thin projections (Figure 4A, arrowhead). This morphology is associated with microglia in a resting state. In contrast, microglia that resided at the RPE/ROS margin exhibited a more rounded morphology with fewer projections (Figure 4A, white chevron). This morphology is associated with microglia in an intermediate/activated state, but not actively phagocytosing debris. We quantified microglia area in 0 h CLL control retinas and compared microglia with resting morphologies in the inner retina to microglia with rounded morphologies in the RPE/ROS margin and found no statistical difference (Supplementary Figure S6A, *p* = 0.0524).

In the CLL model, microglia remained at near baseline levels within the first 36 hpl (Figures 4B, Q). Starting at 72 hpl, we observed an increase in the presence of microglia with a rounded morphology at the outer margin of the degenerating ROS (Figures 4D–H, Q). Compared with baseline controls, this increase in number was moderate (*p* = 0.011 at 10 dpl), but sustained throughout the time-course, and by 14 dpl, microglia with a rounded morphology formed a single line at the RPE/ROS interface (Figure 4G). In contrast, in the AL model, large numbers of microglia with ameboid morphology appeared at 36 hpl (Figure 4K, arrowhead), and the peak of microglia accumulation occurred at 72 hpl (Figures 4L, Q). The morphology of the microglia in the outer retina in the AL model was nearly all ameboid, and the response was nearly 4-fold greater at peak in the AL model vs. the CLL model (Figure 4Q, *p* < 0.0001). This ameboid morphology is characteristic of active phagocytosis, which is consistent with these microglia clearing the debris field of dead PRs at these time-points. We quantified microglia area at 72 hpl in the CLL and AL models and found that microglia in the AL model with amoeboid morphology had a significantly greater area than microglia with a rounded morphology in the CLL model (Supplementary Figure S6B, *p* < 0.0001). These data are consistent with a greater phagocytosing activity in the microglia with an amoeboid morphology in the AL model. Finally, at later time-points, microglia slowly decreased in number in the AL model, but did not return to baseline control levels by 28 dpl (Figure 4Q).

As an initial comparison of the transcriptional kinetics of the microglial infiltration in the AL and CLL models, we plotted the gene expression data for two microglial associated genes, *mpeg1.1* and *apoeb* (Butovsky et al., 2014; Ferrero et al., 2020; Thiel et al., 2022). In the CLL model, these genes showed a slow, prolonged wave of elevation by ∼72 hpl, a peak at 10 dpl, and then a slow decline through 28 days, trending towards baseline expression levels (Figures 4R, S; blue line). This expression profile correlated with the peak of activated microglia at 10 dpl in the CLL, and the subsequent plateau in these numbers through 28 dpl. This was in contrast to the AL model, which exhibited a rapid immune response as the PRs were destroyed, with *mpeg1.1* peaking at 24 hpl and *apoeb* at 72hpl, followed by a sharp decrease in expression of both genes towards baseline levels (Figures 4R, S; gray line). In both models, we observed an apparent transcriptional resolution of the activated microglial-associated genes, despite the removal of stimulus in the CLL model. Interestingly, this transcriptional resolution was somewhat in contrast to the moderately elevated numbers of microglia still present at the conclusion of the 28-day time-course.

### 3.6 Distinct responses in Müller glia gliosis and proliferation in the CLL and AL models

**FIGURE 5:**
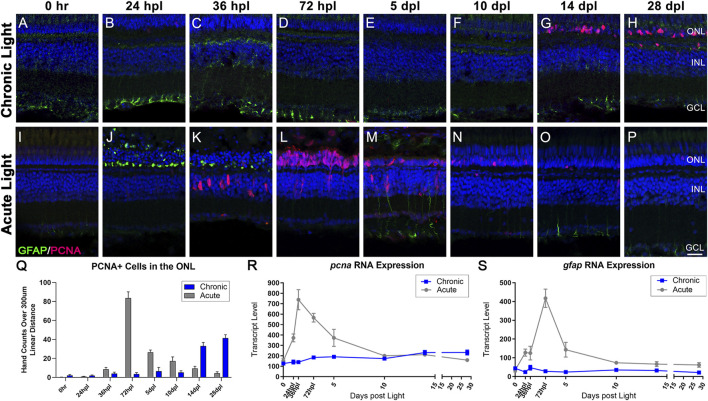
Distinct responses in Müller glia gliosis and proliferation in the CLL and AL models. Müller glia (MG) intermediate filaments were immunolabelled with anti-GFAP antisera (GFAP; green) and cell cycle re-entry was demonstrated with anti-PCNA antisera (PCNA; red). Nuclei were stained with TO-PRO-3 (blue). **(A–H)** The CLL model revealed minimal evidence of MG gliosis, localized to the inner retinal end feet. PCNA+ cells were minimal until 14 dpl, which demonstrated a significant increase in PCNA+ cells in the ONL that increased through 28 dpl. **(I–P)** Classic MG-mediated retinal regeneration in response to AL damage revealed an early increase in GFAP intensity in the ONL MG end feet at 24 hpl, and a second GFAP peak at 5 dpl. PCNA + cells resulting from MG division appeared in the INL at 36 hpl, which was followed by peak MGPC proliferation in the ONL at 72 hpl **(Q)** PCNA-positive nuclei quantified by hand-count over a 300 µm linear distance demonstrated the robust stem cell proliferation in the AL model (72 hpl-5 dpl; *p* < 0.0001), and late accumulation of PCNA + cells in the CLL model (14–28 dpl *p* < 0.0001). **(R,S)** Gene expression changes in *pcna* and *gfap* in CLL vs. AL damage models displayed as transcript pseudocounts from 3′mRNA-seq of individual retinas. **(R)** The transcriptional response of *pcna* followed the histological observations, with a strong peak in the AL model at 72 hpl vs. a slow mild increase in expression in the CLL model. **(S)** The *gfap* expression profile highlighted the robust gliotic response in the AL model in contrast with the absence of a significant response in the ALL model. (ONL = outer nuclear layer, INL = inner nuclear layer, GCL = ganglion cell layer; scale bar in panel P = 5 µm).

Using a limited number of time-points, our previous work suggested that MG do not undergo gliosis in the CLL model (Turkalj et al., 2021). Consistent with this report, we observed that MG in CLL did not significantly upregulate the expression of the intermediate filament GFAP, which is a hallmark of reactive gliosis (Figures 5B–H, Q) (Thomas et al., 2016; Ranski et al., 2018). In addition, our transcriptome studies confirmed no significant changes in *gfap* expression in the CLL model (Figure 5S). These findings were both in contrast to the AL model (Figures 5I–P), wherein MG first exhibited a gliotic response with a consolidation of GFAP in the ONL at 24–36 hpl (Figures 5J, K). This was followed by a more global MG gliosis at 5 dpl, with GFAP expression extending beyond the end feet into the inner retina (Figure 5M). These two waves were also represented transcriptionally, with a significant increase in *gfap* expression at 24 hpl, followed by a dramatic rise to a larger peak at 72 hpl (Figure 5S).

Next, we compared the proliferation responses in the CLL and AL models. In the CLL model, we confirmed the absence of a robust MG proliferation response in the INL throughout the time-course (Figures 5A–H). Instead, we observed a marked and significant increase in a subset of cells within the ONL that expressed the proliferative marker PCNA at 14 dpl, and continued at 28 dpl (Figures 5G, H; *p* < 0.0001). Likewise, *pcna* gene expression showed a mild, but steady increase starting at 3 dpl, which continued through the end of the time-course (Figure 5R). Based on morphology and location, it is highly likely that these PCNA + cells in the ONL represented rod precursors. During constant neurogenesis of the adult zebrafish retina, rod PRs are added to the retina in an injury-independent manner (Stenkamp, 2011). As clusters of MG-derived progenitor cells (MGPCs) were never observed in this model, we hypothesized that the CLL model triggered an upregulation of this injury-independent neurogenesis mechanism. In support of this, we found evidence of both rod precursor proliferation within the ONL and migration of single PCNA + nuclei from INL to the ONL at 14 dpl in the CLL model (Supplementary Figure S7). In contrast, in the AL model, MG reentered the cell cycle at 36 hpl (Figure 5K) and generated a daughter cell that amplified to become a pool of multipotent MGPCs. This robust response was observed transcriptionally with a dramatic increase in *pcna* expression at 24–36 hpl (Figure 5R). The MGPCs then migrated to the ONL at 72 hpl (Figures 5L, Q), and from 5 to 10 dpl differentiated into new PRs (Figure 2N, 3N) and PCNA positivity decreased throughout the remainder of the time-course (Figures 5N–Q). Collectively, these data suggest very distinct responses from MG in that AL and CL models.

### 3.7 Comparative transcriptomic analysis of degeneration and regeneration models reveals inverse patterns of genes implicated in photoreceptor function and development

**FIGURE 6:**
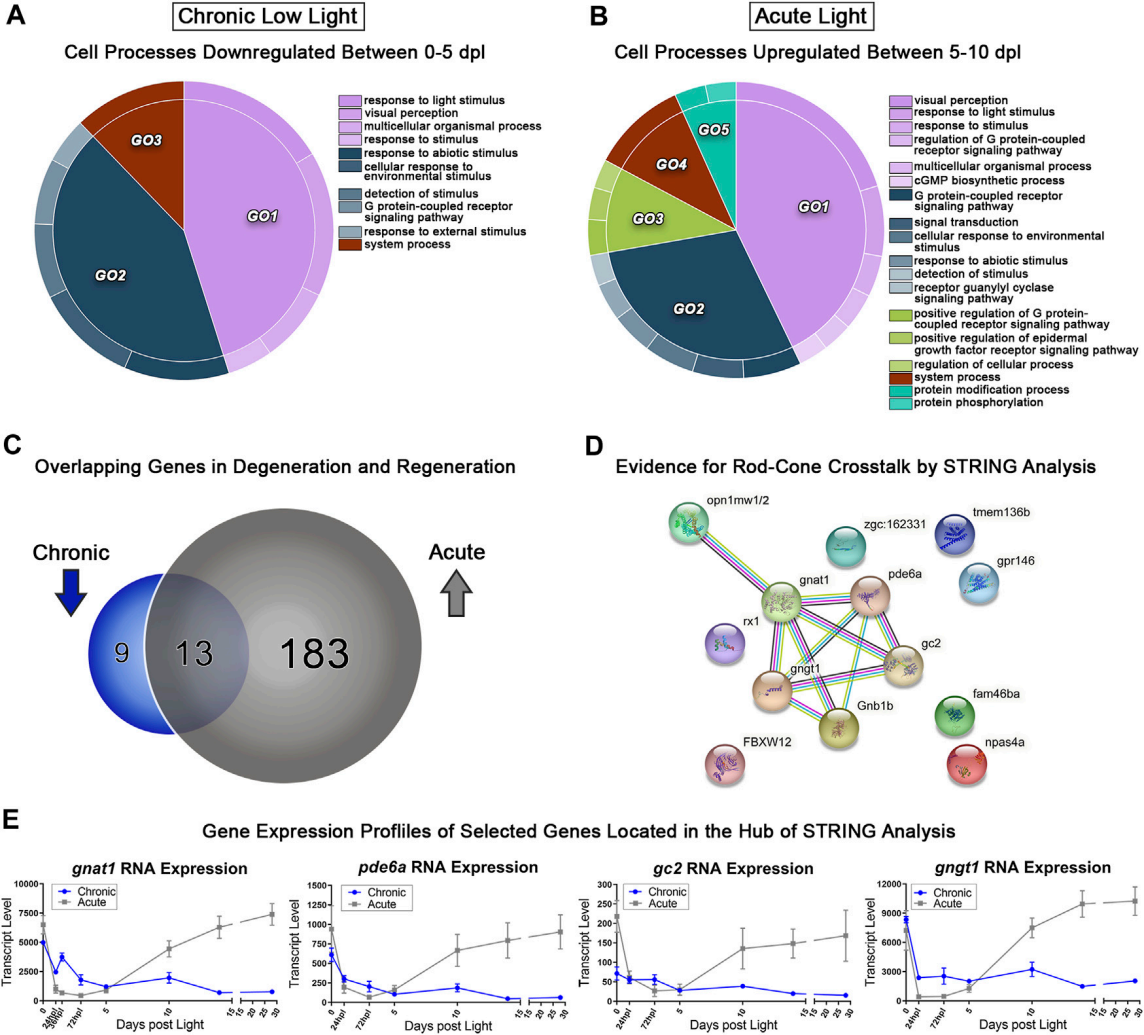
Comparative transcriptomic analysis of photoreceptor degeneration and regeneration. **(A)** Gene ontology (GO) analysis was performed on all downregulated differentially expressed genes (DEGs) in the 0–5 dpl CLL analysis with a fold change ≥2 (FDR<0.05). Redundant terms were consolidated using REVIGO, and visualized using the CirGO visualization software. **(B)** The same GO analysis and visualization was performed for all upregulated genes in the 5–10 dpl window of PR differentiation in the AL model with a fold change ≥2 (FDR<0.05). **(C)** Visual representation of the gene targets identified by the input cut-offs described above, with 22 genes downregulated in the CLL group, 196 upregulated in the AL group, and 13 genes duplicated in both groups, representing inverse transcriptional landscapes during degeneration and regeneration processes. **(D)** The 13 overlapping genes were input into the STRING platform to probe for known genetic interactions, revealing several rod-associated genes interacting with cone opsins during these two processes. **(E)** Transcript pseudocounts for 4 selected genes related to visual function were plotted for the entire 28dpl time-course (chronic low light in blue, acute light in grey).

Using transcriptomic data for both AL and CLL models, we probed for genes corresponding early PR degeneration in CLL (0–5 dpl) and PR regeneration in AL (5–10 dpl). We hypothesized that critical PR genes that were significantly downregulated during sustained degeneration in the CLL damage paradigm, would overlap with a subset of genes that were significantly upregulated during regeneration in the AL model. Pairwise analyses between the 0–5 dpl CLL samples and the 5–10 dpl AL samples was performed, and genes with ≥2-fold decreased expression in 0 vs. 5 CLL (FDR<0.05) and ≥2-fold increased expression in 5 vs. 10 AL were categorized by gene ontology (GO) analysis using the GOrilla platform (Eden et al., 2007; Eden et al., 2009). REVIGO was used to consolidate redundant terms, and CirGO software was used to visualize the output in 2D pie chart format (Figures 6A, B) (Supek et al., 2011; Kuznetsova et al., 2019). The majority of differentially expressed genes (DEGs) in both the 0–5 dpl CLL and 5–10 dpl AL datasets were related to phototransduction (light purple GO1 in Figures 6A, B), with categories such as “response to light stimulus” and “visual perception.” The next largest GO term, related to a cellular response to an external stimulus, was also shared between the datasets (slate blue GO2 in Figures 6A, B). Next, when we directly compared the genes within the AL upregulated group (grey) and CLL downregulated group (blue), we found 196 genes in the former category and 22 in the later (Figure 6C). Remarkably, 70% of the CLL downregulated genes were also in the AL upregulated gene set (Figure 6C), confirming our original hypothesis. The 13 genes that overlapped in these datasets included the dominant cone cell opsin, *opn1mw2*, genes known to be involved in rod phototransduction, such as *gnat1,* and *pde6a* (Sun et al., 2018), and a transcription factor known to play a role in retinal development, *rx1* (Figures 6C, D). Interestingly, STRING analysis of the 13 overlapping genes pointed to a relational cross-talk between rod and cone PRs in these models (Figure 6D) (Szklarczyk et al., 2015). We found that a hub of rod-specific phototransduction genes (*gnat1*, *pde6a*, *gc2*, and *gngt1*) have an association with the medium wave-length cone opsins (*opn1mw1* and *opn1mw2*) (Figure 6E), potentially indicating a connection between rod and cones in these time windows.

**FIGURE 7:**
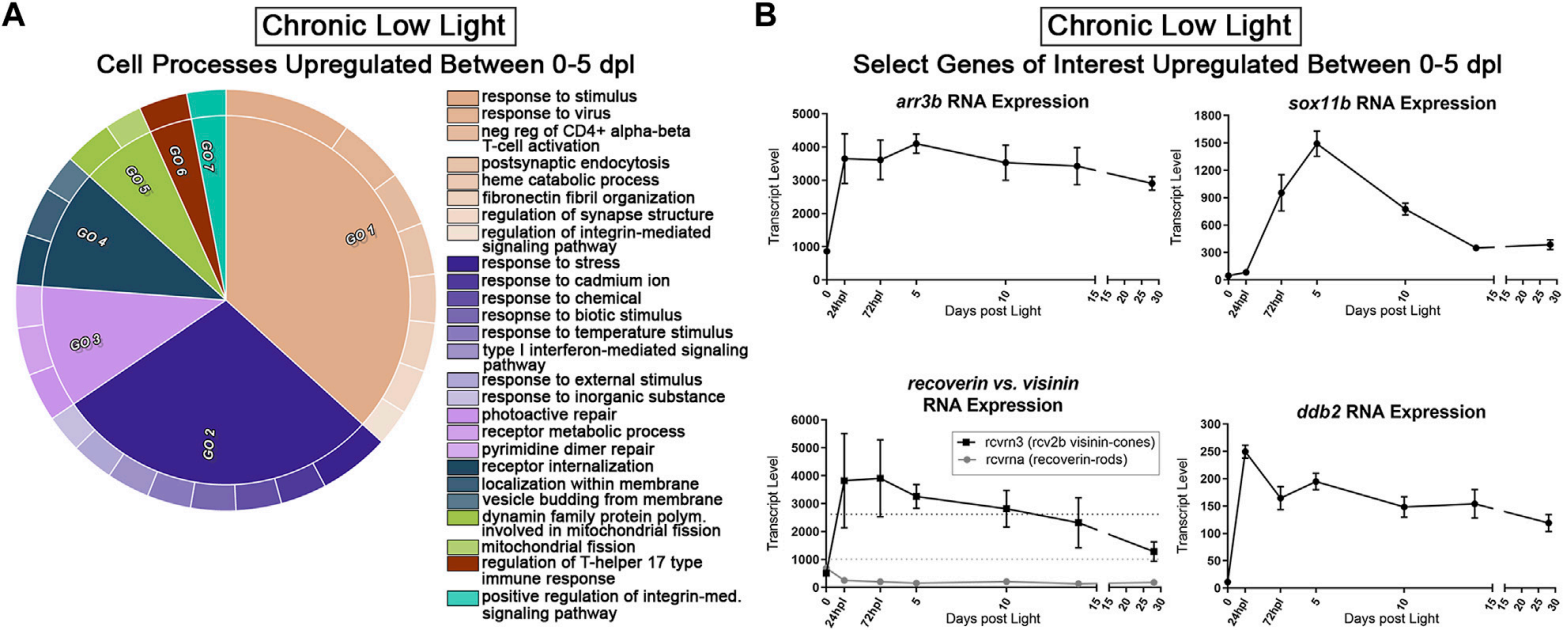
Transcriptomic analysis of early PR degeneration in the CLL Model. **(A,B)** Pairwise comparisons were performed on the 3′mRNA-seq datasets for 0 h and 5 dpl CLL model, representing a window of PR degeneration. **(A)** Gene ontology (GO) analysis was performed on all upregulated differentially expressed genes (DEGs) with a fold change ≥2 (FDR<0.05). Redundant terms were consolidated using REVIGO, and visualized using the CirGO visualization software. Common GO themes identified represent visual system, cell signaling, and immune system processes. **(B)** Select 3′mRNA-seq pseudocount graphs for genes that represent putative PR survival mechanisms.

Next, we probed the CLL dataset for genes with a *positive* fold-change ≥2 (FDR<0.05) from 0 to 5 dpl. We hypothesized that these genes represent pro-survival networks for rod and cone PRs. The top two GO categories for this group of genes included classifications of “response to stimulus,” “response to stress,” and “regulation of synapse structure” (beige and dark purple GOs, Figure 7A). Various other GO categories related to cell surface signaling and immune system processes (Figure 7A). We also found significant changes in select genes known to encode proteins that play roles in visual perception, DNA repair, and eye development (Figure 7B). For example, the *arrestin3b* gene (*arr3b*), encodes a G-protein coupled receptor binding protein required for the termination of cone phototransduction. Similar to GNAT1 in rods, ARR3 translocates from the outer segment to the inner segment of cones following bright light stimulation (Zhang et al., 2003), supporting a role for these proteins in light-dark adaptation. The *ddb2* gene encodes a p53-associated protein involved in global DNA repair following UV light damage (Hwang et al., 1999). The *sox11b* transcription factor is critical in central nervous system development, including retina and lens formation (Wurm et al., 2008; Jiang et al., 2013; Anand and Lachke, 2017) where it plays a role to maintain proper levels of Hedgehog signaling during ocular morphogenesis (Pillai-Kastoori et al., 2014). Finally, *rcvrn3* (*visinin*) encodes a cone-specific recoverin that modulates the recovery phase of the PR response to light stimulation, leading to their prolonged life. This *visinin* gene (*rcvrn3*), however, is unique to zebrafish and some species of birds and amphibians and we found that it had an inverse expression pattern in the CLL model relative to its rod counterpart, *rcvrna* (recoverin) (Figure 7B) (Lamb and Hunt, 2018). Collectively, this dataset offers the field a new set of genetic networks associated with chronic PR degeneration and survival.

### 3.8 Photoreceptors partially recover when the CLL stimulus is removed

**FIGURE 8:**
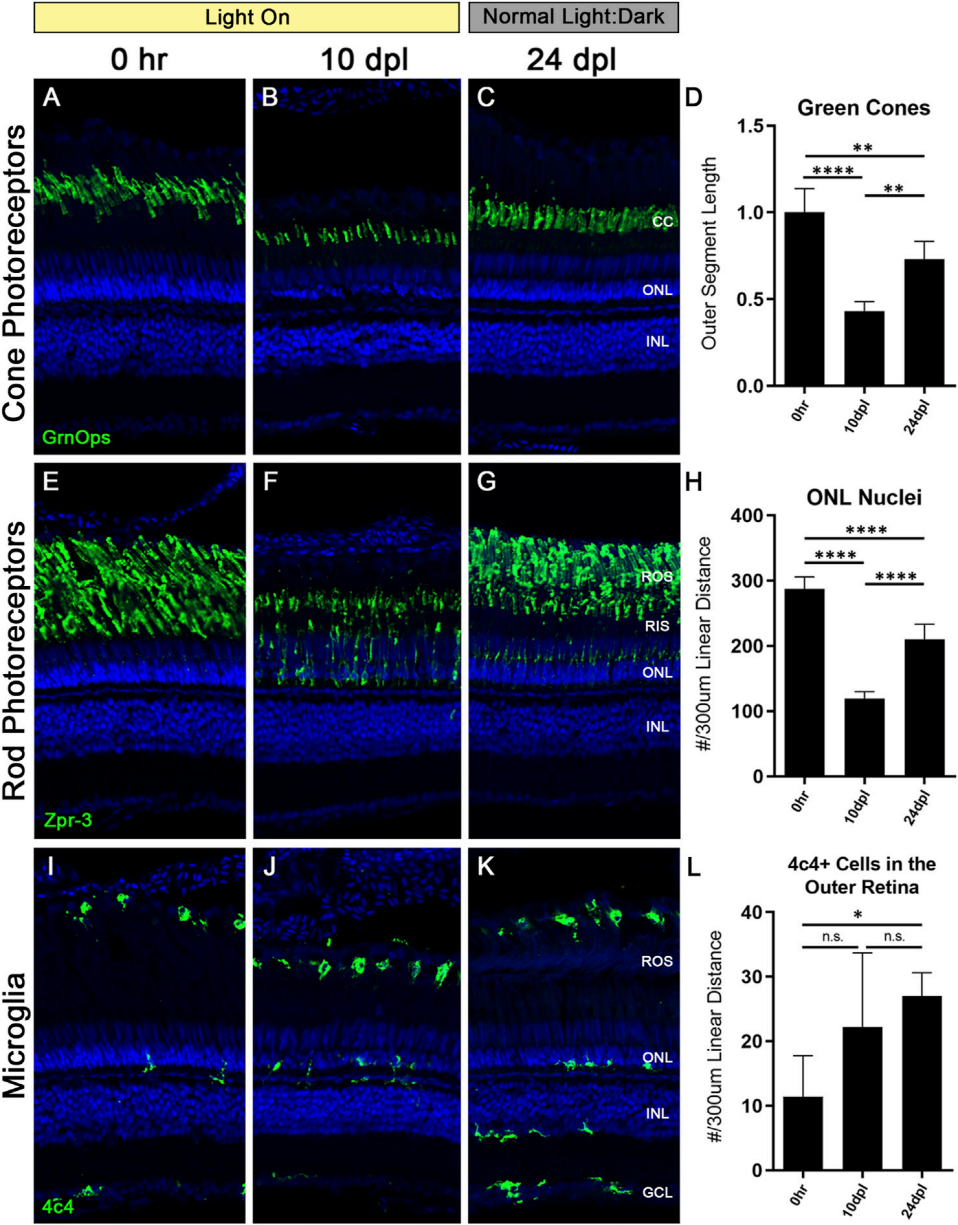
Truncated photoreceptor outer segments recover when zebrafish are transferred back to normal light:dark conditions. Zebrafish were subjected to the CLL lesion protocol for 10 days, then returned to normal light:dark housing conditions and allowed to recover for 14 days. After the 14-day recovery period, retinas were harvested at 24 days post initial light (dpl). **(A–C)** Retinal sections were immunolabelled with anti-Green Opsin (green) labelling green cone PRs. Significant truncation of cone outer segments (OS) was observed at 10 dpl, and following the 14-day recovery, cone OS length was partially restored. **(D)** Green cone OS length was quantified using ImageJ (*n* = 5–6). At 10 dpl of CLL exposure, OS length was reduced by >50% (*p* < 0.0001). Partial recovery of OS length was achieved to ∼75% of baseline levels (*p* < 0.002). **(E–G)** Rod PRs were immunolabelled with zpr-3 marking rhodopsin (green). **(F)** At 10dpl, we observed significant truncation of the ROS and internalization of the zpr-3 signal. **(G)** Following the 14-day recovery under normal light conditions, ROS presented morphological recovery and OS length was partially restored. **(H)** ONL nuclei were quantified by hand count over a 300 µm linear distance. Following significant loss of ONL nuclei at 10dpl (*p* < 0.0001), after retinas recovered for 14 days, ONL nuclei counts were restored up to ∼75% of the 0 h baseline (*p* < 0.0001). **(I–K)** Microglia in retinal sections were labeled with a 4c4+ antibody (green). 4c4+ cells clearly reside at the outer margin of the ROS at every stage of the protocol. **(L)** Hand count of 4c4+ cells present in the outer retina PR-layer over a 300 µm linear distance reveals an increase in microglia after the onset of the light exposure, despite removal of the light stimulus (*p* < 0.01 at 28 dpl). (CC = green cone cell outer segments, ONL = outer nuclear layer, INL = inner nuclear layer, ROS = rod outer segments, RIS = rod inner segments, GCL = ganglion cell layer).

At 10 dpl in the CLL model both rod and cone OS were truncated by 40%–60%; however, the cone cells were still present, whereas a loss of some rod PRs was evident based on the thinning ONL (Figures 2, 3). This predilection for damaging rods over cones is similar to what is observed in some human retinal degenerative diseases, such as age-related macular degeneration (Curcio et al., 1996). Unlike the disease state, which is not reversible, the CLL model can be discontinued at any time, allowing us to test whether this chronic stress on PRs is reversible. Specifically, we asked whether the discontinuation of the CLL stimulus would allow for recovery of truncated rod and cone OS and the restoration of rod nuclei. To test this, we subjected the animals to CLL for 10 days, and then returned the remaining unharvested animals back to their normal light:dark housing conditions for 2 weeks (Figures 1, 8). Eyes were harvested for immunohistochemical analysis at 0 h, 10 dpl, and after a 2-week recovery period at 24 dpl (Figure 8).

We observed a significant recovery of both rod and cone OS (Figures 8C, G). Green cone outer segments recovered to ∼75% their original length (Figure 8D), but lacked the classic cone-shaped morphology, with the tips of the outer segments having a frayed appearance (Figure 8C). Rod outer segments also recovered to ∼75% of their length; however, zpr-3 staining was still evident in the RIS. ONL nuclei, which are primarily rod PRs, also recovered to ∼73% of normal from the reduction at 10 dpl (Figure 8H). Finally, despite removal of the light stimulus, we observed a significant number of activated microglia present in the subretinal space near the RPE/ROS boundary compared with undamaged control retinas (Figures 8I, L; *p* < 0.01). Of note, microglia in the inner retina remained in a resting/ramified morphological state, similar to control retinas (Figures 8I, K). Future studies will be aimed at determining the role of these microglia in CLL damage conditions and recovery.

## 4 Discussion

For over two decades, the adult zebrafish retina has been extensively studied due to its remarkable capacity to regenerate upon significant acute insult (Fausett and Goldman, 2006; Bernardos et al., 2007; Fimbel et al., 2007; Thummel et al., 2008b). These studies have contributed to our fundamental understanding of the stem-cell potential of MG, PR developmental pathways in the adult retina, and immune cell roles in this process. With the advent of recent low-cost, low-input RNA sequencing technologies like 3′mRNA-seq, studies of the transcriptional consequences of retinal damage have become more accessible to a broader range of researchers. Towards this end, we previously reported a transcriptional and morphological characterization of the full 28-day time-course of retinal regeneration in the AL model (Kramer et al., 2021). However, the AL model does not accurately reflect the slow PR degeneration observed in numerous hereditary retinal degenerative diseases, or in late onset diseases of the aging retina such as age-related macular degeneration. As such, we sought to further investigate our recently-described CLL model (Turkalj et al., 2021) and directly compare it to the AL model. This comparative analysis revealed a number of novel findings in our CLL model, including a differential sensitivity of rods and cones, a “switch” in green opsin paralogue expression, a lack of overt MG gliosis or proliferation, and data showing that PRs can recover if the CLL damage is discontinued. Comparative transcriptomic analysis also revealed a subset of genes that are likely critical in both models, and candidate pro-survival genes for future functional tests.

It is well established that cone PRs are more resistant to broad spectrum light damage than rod PRs, which is a consequence of their functional specialty (Korenbrot and Rebrik, 2002). However, there is still a need to better understand how cone PRs respond to stress in models permissive to regeneration, with the goal of harnessing the mechanisms of these stress response pathways for targeted therapeutic approaches in human retinal degenerative diseases. We observed that cone PRs in the CLL model exhibited rapid truncation of their outer segments to ∼50% of their baseline length at 24 hpl, and that this truncation was maintained throughout the entire time-course (Figures 2B, Q; Supplementary Figure S1). Maintenance of the truncated outer segments was likely aided by an increase in cone opsin expression. For example, after falling nearly to zero transcripts at 24 hpl, the green cone opsin (*opn1mw2*) began an upward trajectory of expression, despite the animals remaining in the light stimulus for the entire 28-day time-course (Figure 2R). This observation was consistent for all cone opsins (Figure 2R; Supplementary Figures S1K, V), and even reached full recovery to the 0 h baseline for UV opsin (*opn1sw1*; Supplementary Figure S1K). Interestingly, we also observed a “switch” in green opsin paralog expression with the recruitment and significant upregulation of *opn1mw1*, the green opsin paralog that normally has the lowest number of transcripts expressed (Figure 2R). We hypothesize that this switch and the slow increase of expression in all the opsins represents a mechanism to adapt to conditions of chronic stress. Another potential mechanism to protect cones from CLL damage is the gene and protein expression changes we observed to Arrestin. Arrestins function in the visual system to shut off phototransduction (Renninger et al., 2011; Deming et al., 2015). In the CLL model, we found a tripling of expression of cone arrestin (*arr3a*) transcripts immediately following light onset (Supplementary Figure S2I). In addition, we noted a shift in Arrestin3a localization (as determined by zpr-1 immunolocalization) from the perinuclear region to the OS during the early time-points following light onset (Supplementary Figures S2B, C). This translocation is consistent with mammalian models of light exposure (Zhang et al., 2003; Coleman and Semple-Rowland, 2005) and our previous characterization of this CLL model (Turkalj et al., 2021), and may represent an attempt to dampen the phototransduction signal in the OS and reduce phototoxicity.

We found an interesting pattern of expression in a set of phototransduction associated DEGs which are relatively understudied in zebrafish retinal degeneration and regeneration (Figure 7B). These two genes, *rcvrna* and *rcvrn3,* encode two different, but related, classes of proteins termed Recoverins and Visinins, respectively. Recoverins are expressed in nearly all vertebrate retinal PRs, and represent a class of Ca^2+^ binding proteins that modulate the recovery phase of the PR response to light stimulation. Levels of Ca^2+^ are higher during conditions of low light, and in this state, Recoverin inhibits activity of Grk1, which slows the phosphorylation of activated rhodopsin, leading to prolonged active lifetime of rhodopsin (Lamb and Hunt, 2018; Hofmann and Lamb, 2023). Visinins serve the same general physiological function in PRs, with two important distinctions: 1) they are expressed in cone PRs rather than rods, and 2) they are not expressed in mammals. Visinins were lost to mammals and cartilaginous fish during evolution, but some species of birds, amphibians, and bony fish such as the zebrafish, have retained these genes (Lamb and Hunt, 2018; Ogawa and Corbo, 2021). Notably, these are all organisms capable of at least some level of retinal regeneration. Interestingly, in the CLL damage paradigm, *rcvrna* (Recoverin a) exhibited ∼2-fold reduction in gene expression from 0 to 5 dpl; in contrast, *rcvrn3* (Visinin) gene expression increased ∼2.7 fold in this same time window (Figure 7B). This stark contrast in rod vs. cone PR expression of these pro-survival phototransduction genes may lend insight into why cones are more resistant to CLL exposure.

Rod PRs showed stark differences to cone PRs in the CLL model, both morphologically and transcriptionally. In contrast to the immediate truncation of OS observed in cone PRs in the CLL model, rod OS exhibited a slow progressive truncation of the outer segments (Figures 3A–H). This truncation was statistically significant at 72 hpl and continued until the OS were nearly absent at 28 dpl (Figure 3H). OS truncation was also accompanied by a loss of ONL nuclei, which are primarily rod PRs (Figures 3H, 8H) (Raymond and Barthel, 2004; Lagman et al., 2015). This loss and rod OS truncation are reflected in the slow and steady decline of *rhodopsin* gene expression throughout the time-course (Figure 3S). Interestingly, however, not all rods were lost. Indeed, we observed an apparent LD_50_ of light exposure for rod PRs in the CLL model, in which we saw a ∼50% reduction in rod nuclei at 10 dpl that held relatively steady through 28dpl (0 h baseline: 287 nuc ±18.7, 28 dpl: 126.8 nuc ±26.2).

We hypothesize that rods are maintained during these later time-points by increasing the rate of rod neurogenesis in the ONL. This is supported by the observation of significant numbers of PCNA + cells in the ONL at later time-points (Figures 5G, H), and the partial recovery of ONL nuclei when the CLL is removed (Figure 8G). This may represent an alternative mechanism for rod PR replacement in this model of light-induced chronic damage. Interestingly, although the MG-mediated regenerative response to acute damage is well characterized, the rod precursor lineage of rod PR replacement is less well described. It is known that rod precursor cells are generated stochastically from slowly cycling MG that undergo asymmetric cell divisions to generate a unipotent rod progenitor in the absence of damage (Stenkamp, 2011). This mechanism is required for normal maintenance of the teleost adult retina, because these animals undergo retinal neurogenesis throughout their lifetime. What has been less studied is whether this mechanism can be co-opted as an alternative approach of rod-specific regeneration. And while this work is the first to show enhancement of rod precursor proliferation in a light damage paradigm, two previous reports have shown similar findings. Using a rod-specific ablation model, Montgomery et al. (2010) showed that if all rod PRs were rapidly killed, the MG-regenerative response was triggered. However, if only a subset of rods were ablated, the MG-response was not triggered; rather, an increase in PCNA + rod precursor cells is observed in the ONL, which is similar to what we observed in the CLL model (Figures 5G, H, Q). Similarly, in a zebrafish P23H model of retinitis pigmentosa, a slow loss of rod PRs triggered a robust expansion of rod precursors in the ONL (Santhanam et al., 2020). It is currently unclear whether this re-purposing of the “injury-independent” rod replacement mechanism occurs by upregulating resident rod progenitor proliferation or by accelerating the cycling of the MG-derived rod precursor mechanism. We found evidence of both putative rod precursor proliferation within the ONL and migration of single PCNA + nuclei from INL to the ONL at 14 dpl in the CLL model (Supplementary Figure S7). Given that we do not find any evidence for MG proliferation in the CLL model nor the generation of large clusters of MGPCs, we support the former hypothesis; however, we cannot exclude the possibility that both mechanisms may be contributing to this “steady state” of rod PR maintenance in the CLL model.

The role of microglia in retinal development and regeneration is a growing area of study (White et al., 2017; Mitchell et al., 2019; Issaka Salia and Mitchell, 2020). In a resting state, microglia typically reside in the plexiform layers of the retina, exhibiting a spindle-shaped, ramified morphology (Nimmerjahn et al., 2005). We also observed microglia with an activated state morphology residing in the subretinal space at the margin of the ROS (Figure 4A). In contrast to the robust accumulation of phagocytic, amoeboid microglia in the AL model, microglia in the CLL model were significantly fewer in number, and appeared to retain an activated, but not amoeboid morphology. We observed these microglia accumulating at the margin of the degenerating rod OS at 10 dpl and persisting through 28 dpl (Figures 4A–H, Q). Fascinatingly, despite the persistent presence of increased numbers of microglia in the retina at the later time-points, after peaking at 10 dpl, the transcript levels of microglia-specific genes *mpeg1.1* and *apoeb* eventually returned to just slightly above baseline (Figures 4R, S). This apparent resolution of the inflammatory response directly correlated with the appearance of PCNA + putative rod progenitors in the ONL at 14 and 28 dpl in the CLL model (Figures 4R,S, 5G, H) and may represent a transcriptional and cellular shift of the retina from a “damage control” state to a modified regenerative repair state. Notably, a similar inflammatory resolution was evident in the AL model, albeit in a more accelerated timeline (Figures 4R, S). The fall in both *mpeg1.1* and *apoeb* transcripts began at 5 dpl in the AL model, immediately following the peak of PCNA + MGPC proliferation in the ONL at 72 hpl (Figures 4R, S, 5L). This observation raises the possibility of a cross-talk between progenitor cells in the ONL and adjacent microglia in order to mediate the resolution of the microglial response.

To further investigate the transcriptional landscapes of these datasets, we chose to perform a deeper analysis comparing the process of PR degeneration in the CLL model, and PR regeneration in the AL model. We chose the 0 h and 5 dpl time-points to represent the process of degeneration in the CLL model because both rods and cones show signs of degeneration within this window (Figure 3). We previously reported that the 5–10 dpl time window in the AL model represents the window of PR differentiation following the proliferation of MGPCs (Kramer et al., 2021). When we performed GO analysis on these two datasets representing degeneration and regeneration of PRs in the CLL and AL models, respectively, we identified several genes involved in visual perception and signaling, including 13 overlapping genes which were significantly downregulated in the CLL degeneration model, and significantly upregulated the ALL model (Figure 6). Based on STRING functional protein network analysis of these 13 overlapping genes, critical components of rod phototransduction such as *gnat1, pde6a, gc2*, and *gntg1* appeared to have an association with medium wavelength cone opsins *opn1mw1* and *opn1mw2*.

It has been well established since the 1970s that rods and cones communicate in the mammalian retina, with some early psychophysics experiments that revealed that rods can counterintuitively contribute to color vision, and there is also strong evidence that cones can carry rod signals (McCann, 1972; Fain and Sampath, 2018). Morphologically, TEM studies reveal that rods and cones interdigitate via telodendral gap junctions (Raviola and Gilula, 1975; Nelson, 1985). Furthermore, short wavelength cones have fewer of these gap junctions, which may be one explanation as to why the medium wavelength opsins were distinguished in our transcriptional analysis in this CLL model (Ahnelt et al., 1990; Schneeweis and Schnapf, 1995).

Evidence for rod-cone crosstalk also exists in the context of retinal disease, particularly in retinitis pigmentosa. Mutations in rhodopsin not only lead to rod death in retinitis pigmentosa, but cones also eventually die (Papermaster and Windle, 1995). Recently, a loss-of-function mutant of a rod-specific *alpha* subunit of *pde6* (*pde6a*) in larval zebrafish revealed that the model recapitulates the human retinitis pigmentosa phenotype, with a preliminary degeneration of rods followed by a subsequent loss of some cones (Crouzier et al., 2021). Experimental insight into rod-cone crosstalk in zebrafish retinas also reveals that an increase in rods can actually protect cones from further degeneration in a cone degeneration model (Saade et al., 2013). Despite evidence for this cross-talk in physiological conditions, exploration of its role in PR degeneration and regeneration is understudied. Our transcriptional evidence indicates that in the CLL model, a genetic network that points to rod-cone crosstalk is significantly upregulated (Figure 6D) and warrants further functional investigation.

Next, we probed the CLL dataset for genes *upregulated* from 0 to 5 dpl, and identified several genes that likely aid PR survival. This includes *ddb2,* which encodes a protein that functions to repair DNA damage in response to UV light. Another gene of interest in this category was *arr3b*, which is the paralog of the *arr3a* gene described above. Finally, we observed strong upregulation of *sox11b,* which encodes a transcription factor that plays key roles during eye development (Usui et al., 2013; Pillai-Kastoori et al., 2014; Chang and Hertz, 2017; Kuwajima et al., 2017). A role for Sox11b in adult zebrafish regeneration was first implicated in a zebrafish model of congenital rod PR degeneration (Morris et al., 2011). More recently, functional knockdown of Sox11b following acute damage to the retina was shown to affect the fate determination of MGPCs (Song et al., 2023). However, it is role in chronic retinal damage models is unknown. Therefore, these genes collectively represent putative targets for functional studies in this model of slow PR degeneration.

Lastly, we chose to investigate whether the damage to the PR OS in the CLL model was reversible (Figure 8). We first exposed animals to the CLL protocol for 10 dpl, and then allowed them to recover for 14 days under normal light:dark conditions. At 10 dpl, cone PRs exhibited a 50% truncation in outer segment length (Figure 8D). ROS also exhibited significant OS truncation, with nearly non-existent OS remaining at 10 dpl; in addition, we observed a 50% reduction in ONL nuclei at this time-point (Figure 8H). Interestingly, cone and rod PRs both recovered to almost exactly 75% of their baseline morphology as measured by OS length (Figures 8D, H). In addition, ONL nuclei also recovered and were likely derived from PCNA + rod progenitor cells that we observed in the ONL of CLL retinas starting at 14dpl (Figure 5G). This finding highlights a new area for investigation into the multi- and unipotent photoreceptor progenitor lineages that can be derived from MG under various damage paradigms. Finally, we observed an unexpected increase in the presence of microglia in the outer retina *after* removal of the stimulus (Figures 8I–L). This is contrary to our expected outcomes, and may indicate a necessity for microglia during this PR recovery process or suggest a sustained inflammatory response following chronic damage.

With this study, we have transcriptionally and morphologically characterized a new model of sub-acute, chronic damage to retinal PRs that did not trigger a MG-mediated multipotent stem cell response. We revealed a dosage of light damage that damaged both cone and rod OS, left cone PR nuclei intact, and reduced rod PR nuclei to 50% of normal numbers. This study also highlights the differential responses to acute and chronic damage in the zebrafish retina, and opens up new avenues for functional investigations of genetic pathways involved in PR survival and recovery.

## Data Availability

The datasets presented in this study can be found in the NCBI Gene Expression Omnibus, accession no: GSE233896, and the associated metadata for this submission can be found in Supplementary Table S2.
