# Supplementary material for: A comparative analysis of gene and protein expression in chronic and acute models of photoreceptor degeneration in adult zebrafish
Source: Front Cell Dev Biol. 2023 Sep 7;11:1233269. doi: 10.3389/fcell.2023.1233269 (PMC10512720; doi:10.3389/fcell.2023.1233269)
Supplement: Supplementary file 3 [file DataSheet1.docx]

Supplementary Material

A comparative analysis of gene and protein expression in chronic and acute models of photoreceptor degeneration in adult zebrafish

**Ashley C. Kramer^1^, Justin Carthage^1^, Yasmeen Berry^1^, Katherine Gurdziel^2^, Tiffany A. Cook^1,3^, Ryan Thummel*^1^**

*** Correspondence:**Ryan Thummel, Ph.D.
rthummel@med.wayne.edu

# Supplementary Data

Mapped reads for the entire dataset can be found in Supplementary Table 1. The datasets presented in this study can be found in the NCBI Gene Expression Omnibus, accession no: GSE233896, and the associated metadata for this submission can be found in Supplementary Table 2.

# Supplementary Figures and Tables

## Supplementary Figures


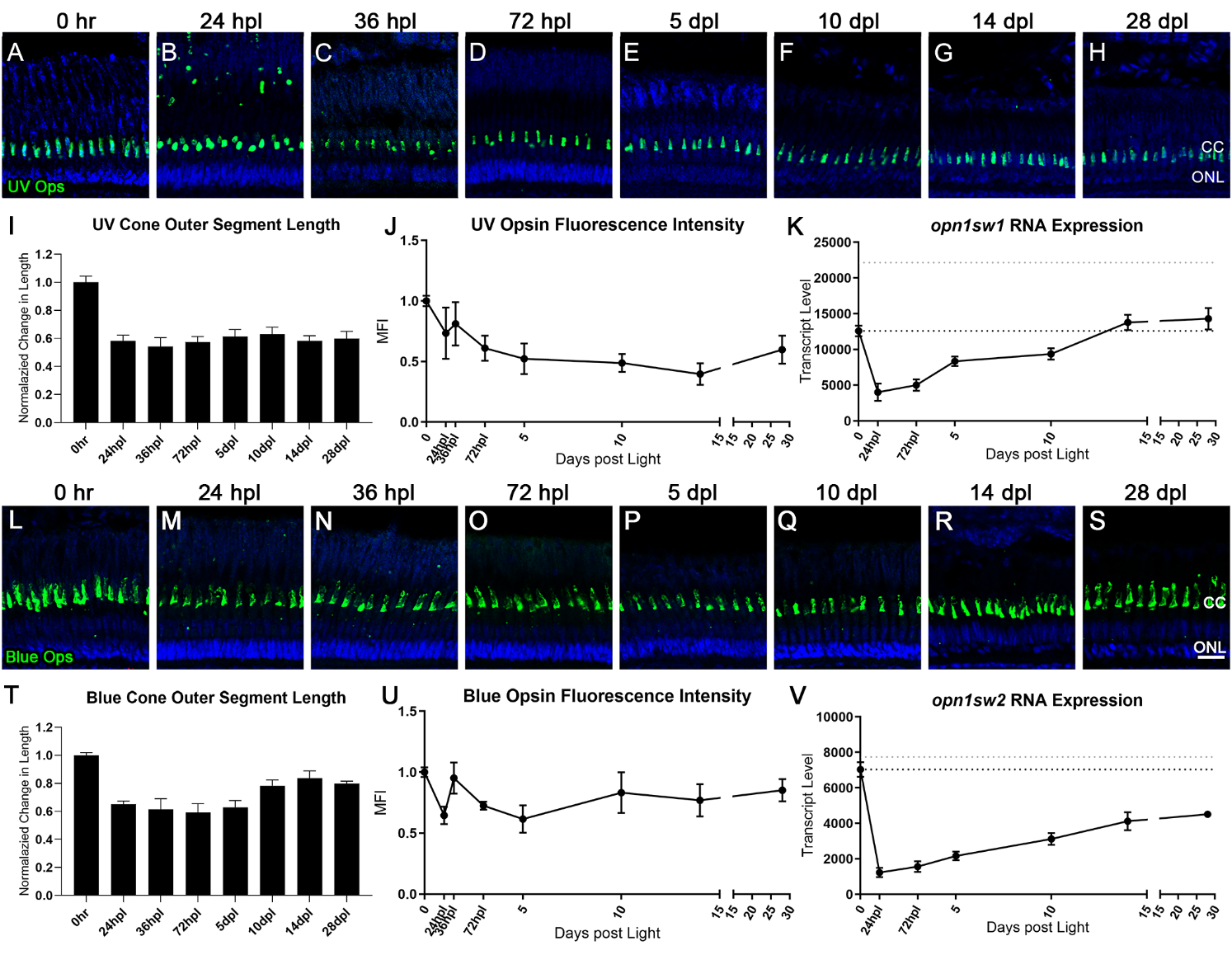


**Supplemental Figure 1. Short wavelength cones exhibit mild truncation in response to chronic low light exposure. (A-H)** Similar to medium wavelength cones in Figure 2, UV cone photoreceptors (UVOps, green; nuclear TO PRO3, blue) showed an early outer segment shedding and sustained truncation over chronic 28-day light exposure. **(B)** At 24hpl, we observed truncation of UV cone photoreceptor outer segments with an immuno-positive debris field in the outer retina. **(C)** At 36hpl, we observed a large reduction of the debris field in the outer retina and continued truncation of UV cone photoreceptor outer segments. **(I)** UV cone outer segment length measured via Image-J, normalized to 0hr baseline. For every measurement 24hpl and beyond, compared to the 0 hr baseline, there was a statistically significant length reduction (p<0.001; n=4-6). **(J)** Relative change from 0hr baseline in ImageJ-quantified UV opsin mean fluorescence intensity (MFI) at each time-point. **(K)** Gene expression changes in UV Opsin (*opn1sw1*) displayed as transcript pseudo-counts from 3’mRNA-seq of individual retinas. The gray horizontal dotted line represents the transcript pseudo-count average baseline value for non-dark adapted, naïve retinas. The black dotted line represents the average 0hr, 1-day dark-adapted baseline gene expression. **(L-S)** Anti-blue opsin antisera (BlueOps; green) was used to immunolabel blue cone photoreceptors and nuclei were stained with TO-PRO-3 (blue). Blue cone photoreceptor outer segments showed a slight truncation but largely remain intact over a 28-day chronic light exposure. **(L)** At 24hpl, we observed a mild truncation of blue cone photoreceptor outer segments. We also noted the absence of a prominent debris field at 24hpl, which was observed with the Green and UV cone opsins. **(T)** Blue cone outer segment length measured via Image-J, normalized to 0hr baseline. There was a statistically significant OS length reduction compared to 0 hr baseline from 24 hpl through 5dpl (p< 0.001; n=4-6). **(U)** Percent change from 0hpl in ImageJ-quantified blue opsin mean fluorescence intensity (MFI) at each time-point demonstrated no statistically significant difference in MFI. **(V)** Gene expression changes in Blue Opsin (*opn1sw2*) displayed as transcript pseudo-counts from 3’mRNA-seq of individual retinas. The gray and black lines indicate parallel baselines as in (I). (CC=UV cone cell OS for (H) and Blue cone cell OS for (S), ONL=outer nuclear layer; scale bar = 5µm).

**
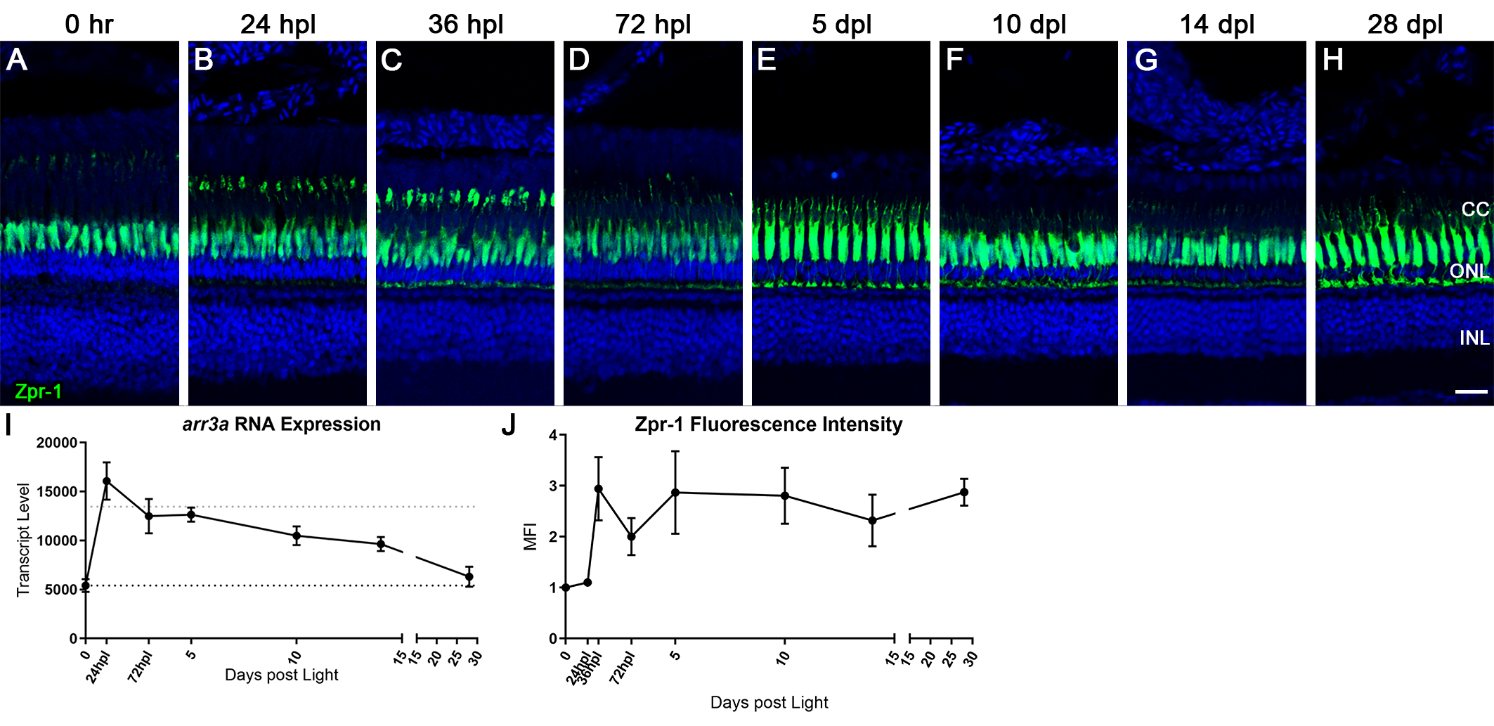
**

**Supplemental Figure 2. Analysis of chronic degeneration of red/green double cone photoreceptors.** Anti-zpr-1 antisera (zpr-1; green) was used to immunolabel red/green double cones, and nuclei were stained with TO-PRO-3 (blue). **(A-H)** Over the 28-day chronic light exposure, the red/green double cones remained in-tact, as demonstrated by the localization of zpr-1 signal to the soma of the cone cells. **(B)** At 24hpl, we observed a new layer of zpr-1 positivity in the region corresponding to cone photoreceptor outer segments which persisted through 36hpl and then disappeared. **(C)** At 36hpl, we observed zpr-1 expression at the base of the ONL in the cone pedicle, which persisted throughout the time-course. **(I)** Gene expression changes in cone arrestin (*arr3a*) corresponding to zpr-1 staining displayed as transcript pseudo-counts from 3’mRNA-seq of individual retinas. The gray horizontal dotted line represents the transcript pseudo-count average baseline value for non-dark adapted, naïve retinas. The black dotted line represents the average 0hr, 1-day dark-adapted baseline gene expression. **(J)** Relative change in ImageJ-quantified mean fluorescence intensity (MFI) relative to 0hr baseline for zpr-1 signal. (CC=red/green cone cell outer segments, ONL=outer nuclear layer, INL=inner nuclear layer; scale bar = 5µm).


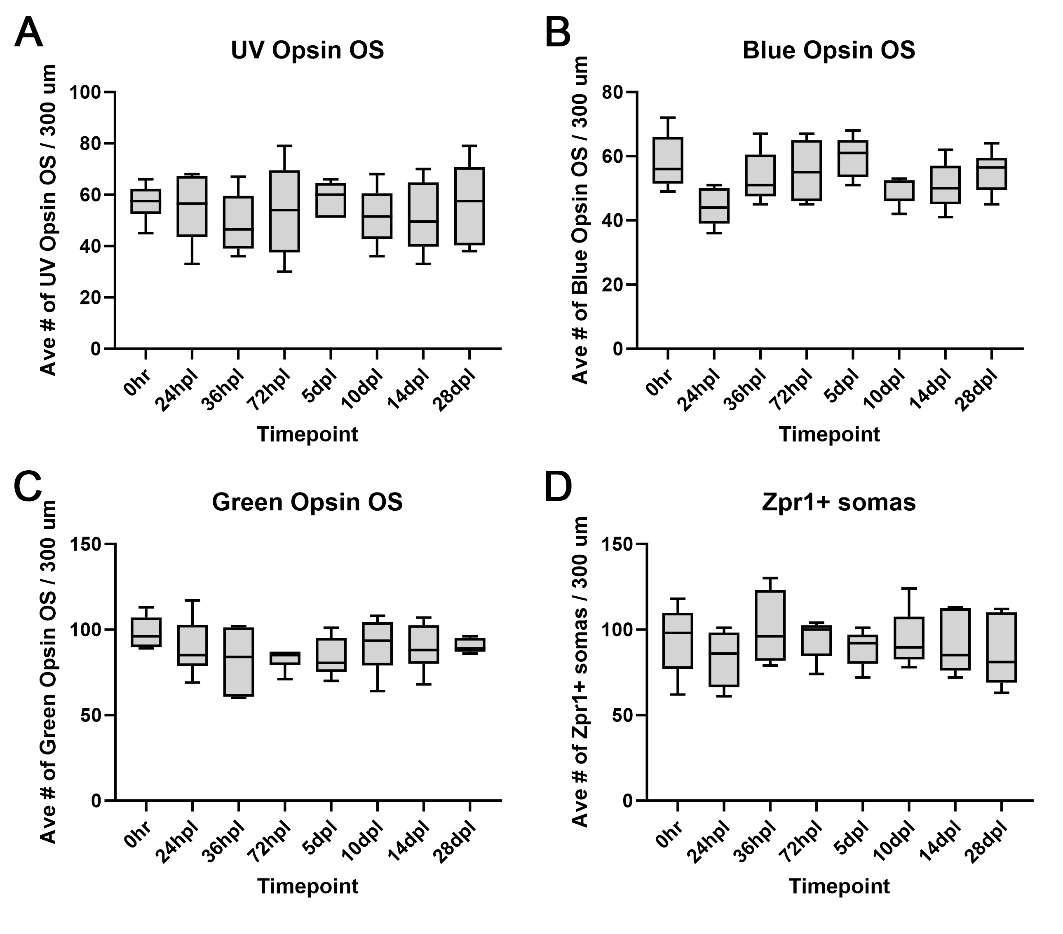


**Supplemental Figure 3. Quantification of cone cells during chronic low light exposure. (A-D)** Histograms of the quantification of outer segments (OS) or cell somas over a 300µm linear distance during the chronic low light time-course. One-way ANOVA was used to determine significant differences between the timepoints throughout the duration of the exposure. No significant differences were observed with regard to UV Opsin OS number (A; p=0.8791), Blue Opsin OS number (B; p=0.0536), Green Opsin OS number (C; p=0.4919), or Zpr1-positive cell somas (D; p=0.7971).


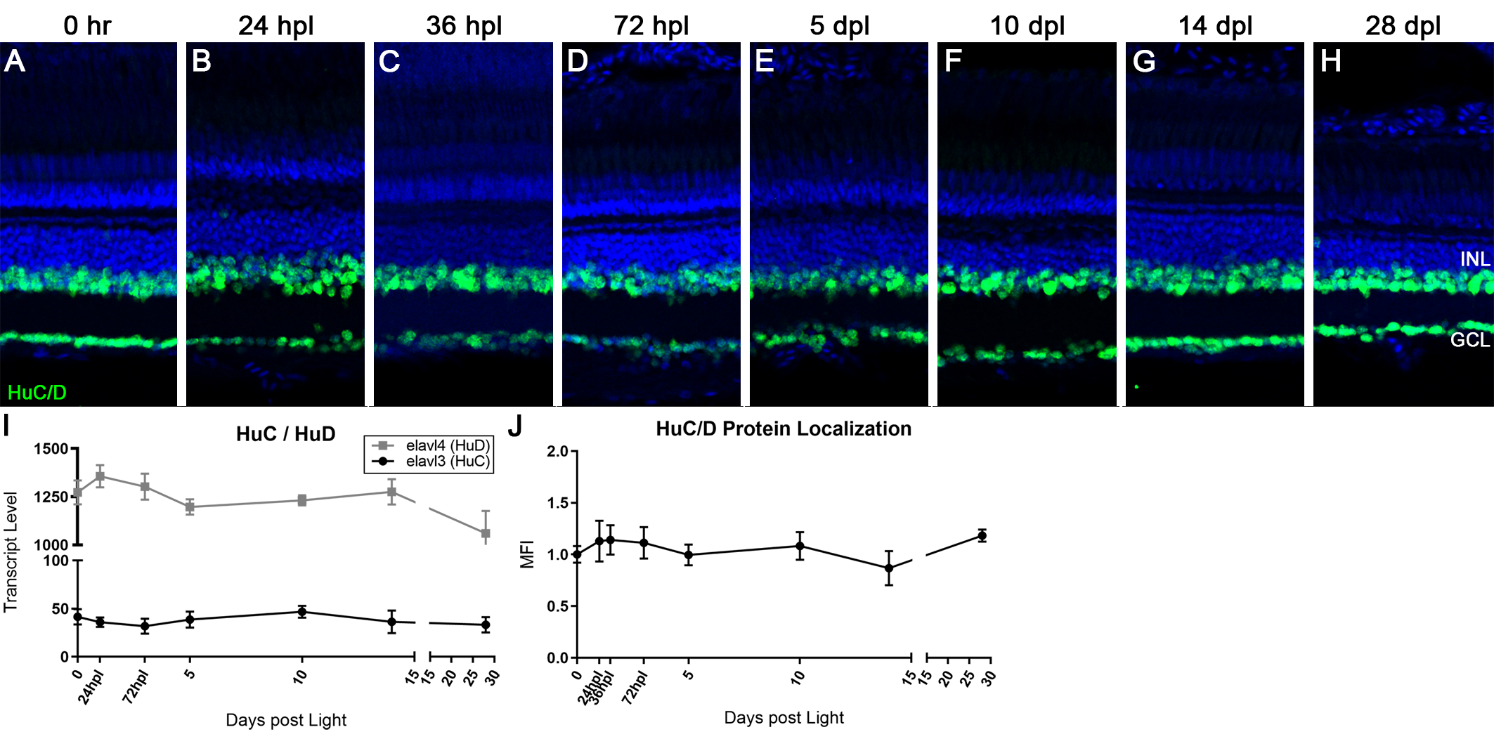


**Supplemental Figure 4. Inner nuclear layer neurons remain intact in response to 28-day chronic light treatment.** Anti-HuC/D antisera (HuC/D; green) was used to immunolabel amacrine and ganglion cells. Nuclei were stained with TO-PRO-3 (blue). **(A-H)** Over the 28-day chronic light exposure, the amacrine and ganglion cell layers appear relatively unaffected in number and location. **(I)** Gene expression changes in HuC (*elavl3;* black) and HuD (*elavl4;* gray) displayed as transcript pseudo-counts from 3’mRNA-seq of individual retinas. The only gene paralogue that exhibited a mild reduction in gene expression throughout the time-course was HuD, with a ~17% decrease in transcript counts from 0-28dpl. **(J)** Relative change in ImageJ-quantified mean fluorescence intensity (MFI) compared to baseline for HuC/D signal remained stable throughout the time-course.


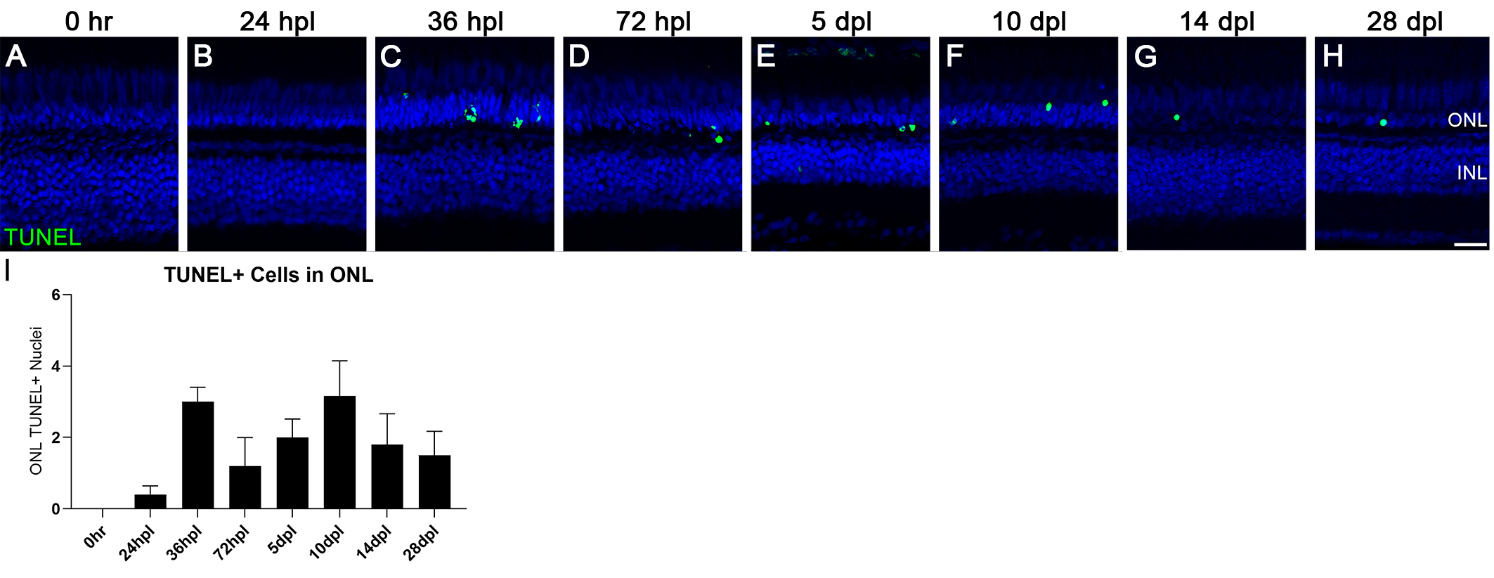


**Supplemental Figure 5. Steady low-level cell death in CLL outer retinas. (A-H)** A terminal deoxynucleotidyl transferase dUTP nick end labelling (TUNEL) assay was performed to detect DNA damage on retinal sections from tissue collected at each of the time-points in the chronic low light model. The incorporated biotinylated dNTPs resulting from the method were visualized using an AlexaFluor 488-conjugated Streptavidin antibody (green). Beginning at 36hpl **(C)** we observed consistent presence of 1-4 TUNEL positive cells in the outer nuclear layer (ONL) indicating the presence of apoptotic cells. **(I)** Graphical representation of hand counts of TUNEL positive nuclei in the ONL over a 300µm linear distance (n=5-6; scale bar in panel H = 5µm).


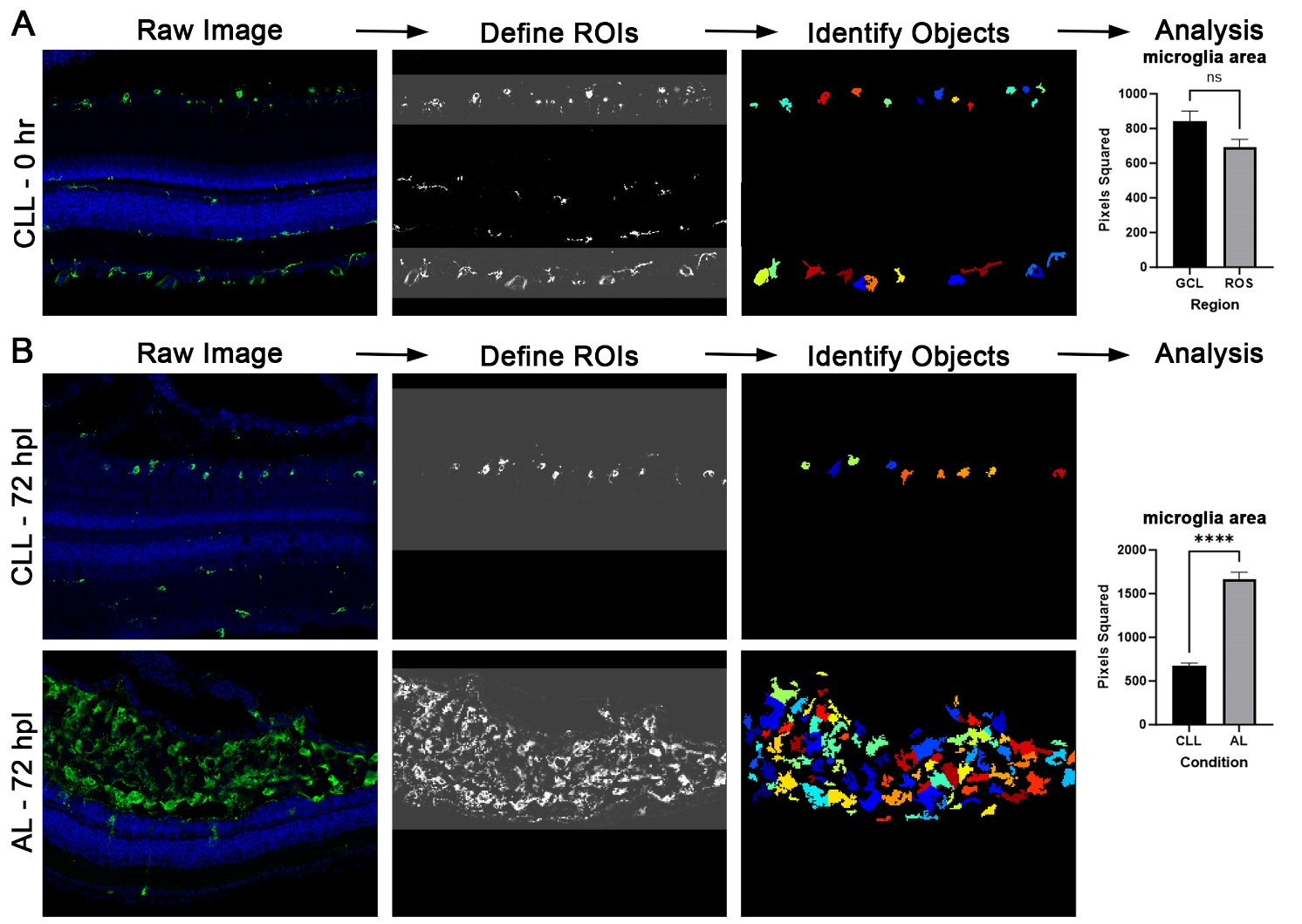


**Supplemental Figure 6. Analysis of microglia morphology in CLL and AL models. (A-B)** CellProfiler Image Analysis Software was used to compare the microglia area in CLL and AL models. Raw images were split into their respective color channels and regions of interest (ROIs) were defined. Modules in the software were utilized to identify separate cells as individual objects and calculate the area of the object. GraphPad Prism 9 was used to determine significant differences between **(A)** microglia area in the RPE/ROS interface vs ganglion cell layer (GCL) in the CLL model at 0 hrs, and **(B)** microglia area in the outer retina in CLL vs AL models at 72 hpl. N=4-6 images per condition. **** indicate p<0.0001.


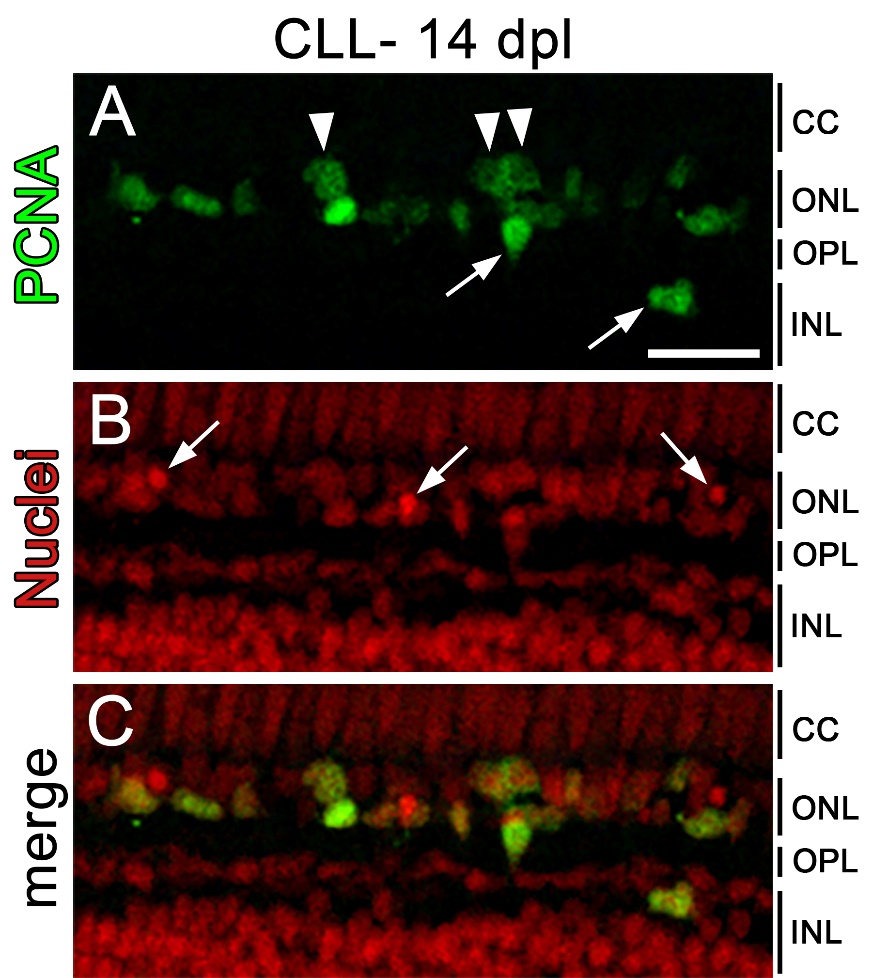


**Supplemental Figure 7. Evidence for rod precursor proliferation and migration in the CLL model. (A).** Proliferating Cell Nuclear Antigen immunolocalization (PCNA, green) shows evidence for rod precursor proliferation within the ONL (arrowheads), as well as proliferation and migration of individual PCNA+ nuclei from the INL to the ONL (arrows). **(B)** Nuclear stain of the same section as shown in Panel A shows continued cell death in the ONL, as evidenced by pyknotic nuclei (arrows). **(C)** A merge of channels shown in Panels A and B. CC = red/green cone cell nuclei; ONL = outer nuclear layer; OPL = outer plexiform layer; INL = inner nuclear layer. Scale bar in panel A = 5µm

*Insert link to Supp Table 1 here*

**Supp Table 1: Raw pseudocount data.** Contains averaged transcript pseudocounts for the six individual biological replicate retinal samples, averages of the biological replicates in a single column on a separate sheet, and pairwise comparisons for 0-5 dpl CLL and 5-10 dpl AL datasets.

*Insert link to Supp Table 2 here*

**Supp Table 2: Metadata sheet for GEO submission.** GEO Submission #GSE233896; NCBI tracking system #24067634.
